# Superior microwave shielding modulation based on rapidly prepared graphene metasurface

**DOI:** 10.1093/nsr/nwaf395

**Published:** 2025-09-17

**Authors:** Pengfei Chen, Xinrui Yang, Yifan Chang, Wei Qian, Huaqiang Fu, Wenxiang Xu, Lin Ren, Zhe Wang, Haoran Zu, Dingsheng Wang, Daping He

**Affiliations:** School of Materials Science and Engineering, Wuhan University of Technology, Wuhan 430070, China; Hubei Engineering Research Center of Radio Frequency Microwave Technology and Application, School of Physics and Mechanics, Wuhan University of Technology, Wuhan 430070, China; State Key Laboratory of Silicate Materials for Architectures, Wuhan University of Technology, Wuhan 430070, China; School of Materials Science and Engineering, Wuhan University of Technology, Wuhan 430070, China; Hubei Engineering Research Center of Radio Frequency Microwave Technology and Application, School of Physics and Mechanics, Wuhan University of Technology, Wuhan 430070, China; School of Materials Science and Engineering, Wuhan University of Technology, Wuhan 430070, China; Hubei Engineering Research Center of Radio Frequency Microwave Technology and Application, School of Physics and Mechanics, Wuhan University of Technology, Wuhan 430070, China; Hubei Engineering Research Center of Radio Frequency Microwave Technology and Application, School of Physics and Mechanics, Wuhan University of Technology, Wuhan 430070, China; State Key Laboratory of Advanced Technology for Materials Synthesis and Processing, Wuhan University of Technology, Wuhan 430070, China; School of Materials Science and Engineering, Wuhan University of Technology, Wuhan 430070, China; Hubei Engineering Research Center of Radio Frequency Microwave Technology and Application, School of Physics and Mechanics, Wuhan University of Technology, Wuhan 430070, China; Hubei Engineering Research Center of Radio Frequency Microwave Technology and Application, School of Physics and Mechanics, Wuhan University of Technology, Wuhan 430070, China; Hubei Engineering Research Center of Radio Frequency Microwave Technology and Application, School of Physics and Mechanics, Wuhan University of Technology, Wuhan 430070, China; Hubei Longzhong Laboratory, Wuhan University of Technology Xiangyang Demonstration Zone, Xiangyang 441000, China; State Key Laboratory of Advanced Technology for Materials Synthesis and Processing, Wuhan University of Technology, Wuhan 430070, China; Hubei Engineering Research Center of Radio Frequency Microwave Technology and Application, School of Physics and Mechanics, Wuhan University of Technology, Wuhan 430070, China; School of Information Engineering, Wuhan University of Technology, Wuhan 430070, China; Department of Chemistry, Tsinghua University, Beijing 100084, China; School of Materials Science and Engineering, Wuhan University of Technology, Wuhan 430070, China; Hubei Engineering Research Center of Radio Frequency Microwave Technology and Application, School of Physics and Mechanics, Wuhan University of Technology, Wuhan 430070, China; State Key Laboratory of Silicate Materials for Architectures, Wuhan University of Technology, Wuhan 430070, China

**Keywords:** electromagnetic shielding modulation, laser-induced graphene, metasurface, rapid preparation

## Abstract

Active manipulation of electromagnetic wave jamming is a critical challenge for next-generation adaptive electronics. Electrical conductivity-tunable porous materials have been developed, but have encountered the challenge of constrained modulation range and large thickness. Herein, we report a method for modulating the shielding efficiency of microwaves based on a micrometer-thick graphene metasurface. The continuous modulation between wave transmission and shielding in an ultra-wide range of 9.66%–99.78% is achieved, due to the remarkable anisotropy of wave-induced electron oscillation. By rotating the metasurface, wherein alignment of the periodically arranged graphene strips with the incident electric field enhances electron oscillations and boosts secondary radiation, significantly improved shielding efficiency is obtained. Notably, the metasurface achieves facile preparation and open-air processability utilizing laser-induced ultrafast kinetics, facilitating its application in advanced smart electromagnetic devices. Finally, we demonstrate its potential in a novel paradigm for data electromagnetic encryption.

## Teaser

A graphene-based metasurface, characterized by facile preparation and open-air processability, has been constructed to efficiently modulate the shielding efficiency of microwaves.

## INTRODUCTION

Electromagnetic shielding materials, including metals, transition metal carbides, and carbon materials, have been widely developed for reducing electromagnetic interference (EMI). However, the inherent fixed physicochemical properties of these materials limit their ability to meet the evolving demands of highly integrated and adaptive smart electronics [1–4]. Therefore, it is highly desired to develop next-generation smart EMI shielding materials with tunable EMI shielding efficiency in response to real-time environmental changes or artificially imposed conditions. This tunability, however, presents challenges in material structure design for synchronously ensuring precise property control and cycle stability.

Five primary regulation strategies have been identified for dynamically modulating EMI shielding efficiency: electrochemical potential [1], mechanical deformation [5–7], rotation [8,9], humidity [10], and temperature [11]. For instance, Han *et al.* employed MXene films to electrochemically drive ion intercalation/de-intercalation with the expansion and shrinkage of MXene layer spacing [1], demonstrating a novel approach for controlling electromagnetic waves. However, the modulation range (94.31%–99.95%) is typically constrained, while the long preparation time (>44 hours) and complex fabrication process of MXene limit the scalability of this strategy. Liu *et al.* prepared a wood-derived carbon/XC-72 nanoparticle aerogel with compression-driven electrical tunability [2]. The compression enables the carbon nanoparticles to establish highly conductive pathways, thereby activating EMI shielding performance. But the excessive thickness of 2–7 cm hinders its application in integrated electronics. It can be seen that the key of the present technical route is to first construct nanostructures and subsequently change nanomaterial blocks’ structure through various external physical fields, thereby affecting conductivity and EMI shielding efficiency. The current challenges of stringent synthesis conditions, prolonged synthesis time, and excessive thickness hinder scalable applications. Thus, it is urgently needed to develop easily-prepared yet large-tunable-range EMI shielding thin film material.

Generally, the EMI shielding efficiency of a material is positively correlated to its electrical conductivity, so highly conductive materials are preferred [12–15]. However, materials capable of superior electromagnetic shielding modulation, achieving wave transmission/shielding (On/Off) switching, requires nearly no/high conductivity in two states. This will put forward even greater requirements for nanostructure construction in thin film [16–20]. As such, constructing an electromagnetic metasurface to achieve tunable reflection and adsorption of electromagnetic waves is an indispensable approach. Both high conductivity and accurate patterning are fundamental, because they jointly determine the modulation capability. However, methods of metasurface preparation, such as screen printing and ink printing, encounter the problem of balancing the viscosity and electrical conductivity of printing inks, thus leading to compromised On/Off switchable performance. To the best of our knowledge, none of the existing EMI shielding thin film materials fully meet the above-mentioned requirements. The design and preparation of such smart EMI shielding material is a notable technological challenge.

Here, we report a laser-induced graphene (LIG)-based smart metasurface with superior EMI shielding modulation capability. The regulation range of EMI shielding efficiency can be broadened to 9.66% (On state)–99.78% (Off state) due to anisotropic wave-induced electron oscillation, which can be activated when the strip-arranged graphene aligns with the incident electric field, thereby enhancing secondary radiation opposite to the incident one. Remarkably, the metasurface can be prepared rapidly in a single step with improved patterning accuracy and a 5  ${\mathrm{ \times }}$  5 cm^2^ surface can be produced in just 5 minutes at room temperature and ambient pressure. The facile fabrication approach enhances its practical applicability. Finally, we demonstrate its substantial role in a novel paradigm for data encoding and encryption. The availability of this metasurface, featuring promising EMI shielding modulation capability, lays the groundwork for future advancements in intelligent electromagnetic devices and systems.

## RESULTS AND DISCUSSION

### Rotation modulated EMI shielding with a LIG-based metasurface

**Figure 1.**
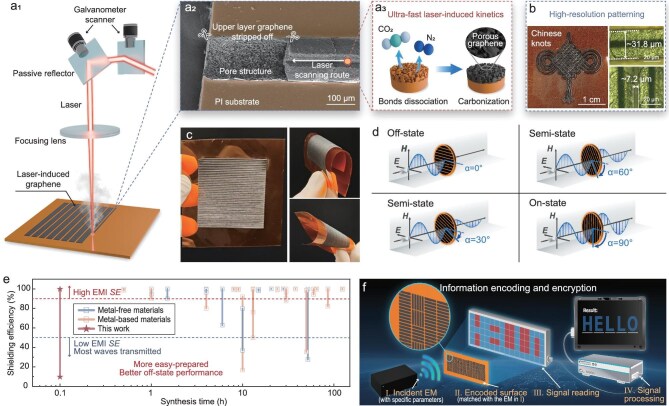
Fabrication, performance and application of the metasurface. (a) Characteristics of LIG technology. (a_1_) Schematic of LIG technology applied on the polyimide (PI) film. (a_2_) SEM image of LIG prepared by one-line laser scanning. (a_3_) Schematic of laser-induced ultra-fast kinetics. (b) Photograph of Chinese-knot-shaped LIG, minimum line width and line spacing. (c) Photograph of the LIG-based metasurface. (d) Schematic illustration of the EMI shielding efficiency modulation. (e) Comparison of synthesis duration and shielding efficiency between reported works and this work. (f) Schematic showing the application of the metasurface on information encoding and encryption.

To realize the rapid preparation of an EMI shielding metasurface under ambient temperature and atmospheric conditions, laser-induced technology was adopted. As depicted in Fig. 1a_1_, a periodically strip-arranged metasurface is designed and fabricated to achieve tunable electromagnetic shielding performance. An infrared laser serves as the light source, converting polyimide (PI) into graphene on the PI film surface through processes of reflection, focusing, and scanning. PI can effectively absorb the infrared laser with a wavelength of 1064 nm [21], inducing instantaneous photochemical reactions (Note S1), including bond dissociation [22,23], carbonization [24], and graphitization [25,26]. After stripping off the upper layer, the gully structure on the surface and inside of the LIG exhibits a distinct porous morphology (Figs 1a_2_ and S1), attributed to the ultrafast release of significant amounts of CO_2_ and N_2_ gases generated during the laser-induced rapid bond-dissociation and carbonization of PI molecules (Fig. 1a_3_). Notably, the high-precision patterning in LIG technology remains a challenge due to the photothermal effect induced by the high-energy pulse, which extends beyond the irradiated area, leading to reduced patterning accuracy, as shown in Fig. S2. Through repeated experimentation (Figs S3 and S4), we enabled the controllable and reproducible achievement of high-precision processing, with a minimum line width and inter-line gap of ∼31.8 μm and ∼7.2 μm, respectively (Fig. 1b). Based on these, complex and high-resolution LIG patterning can be realized, as exemplified by the successful fabrication of a Chinese knot-shaped LIG with dimensions of ∼2.75 × 2.85 cm^2^ (Figs 1b and S5). Compared to previously reported works, this work demonstrates superior patterning accuracy (Table S1 and Fig. S6). This lays a critical foundation for the fabrication of EMI shielding metasurfaces, as the LIG widths and gap widths are pivotal in determining the electromagnetic modulation performance (Note S2).

Figure 1c shows a photograph of the strip-arranged LIG metasurface sample, demonstrating exceptional flexibility while maintaining structural integrity after bending and twisting. This design facilitates the modulation of wave-induced electron oscillation strength and secondary radiation, thereby enabling dynamic control of the interaction between electromagnetic waves and the shielding material. As *α* increases from 0° to 90°, the penetrating electromagnetic field progressively increases in intensity, transitioning through the ‘Off’, ‘Semi’, and ‘On’ states, respectively (Fig. 1d), exhibiting remarkable EMI shielding switching characteristics. The tunable range is a critical metric for assessing dynamic wave control performance, with the surface exhibiting distinctly different electromagnetic responses (wave transmission/shielding) in the On/Off states. In this context, the widest reported tunable range of EMI shielding efficiency (11.5%–99.8%) and shortened synthesis duration of 5 minutes have been achieved (Fig. 1e and Table S2). Notably, synthesis times are reported as the sum of individual process durations, excluding inter-step intervals. While various factors influence synthesis duration, we present these data as a qualitative reference, underscoring the urgency of developing practical, industrially relevant synthesis methods for electromagnetic functional thin films. Shielding efficiency (measured in %) is considered a more appropriate performance metric for On/Off switchable EMI shielding modulation than SE (measured in dB), as SE values, processed mathematically to describe high shielding efficiency (>90%) but fail to intuitively reflect On-state performance (Note S3). Notably, the characteristics of single-step patterning fabrication, easy control, reversibility, and rotation modulated switching facilitate its critical prospect in information coding and encryption (Fig. 1f).

### Laser-focused/defocused-dependent properties of LIG

**Figure 2.**
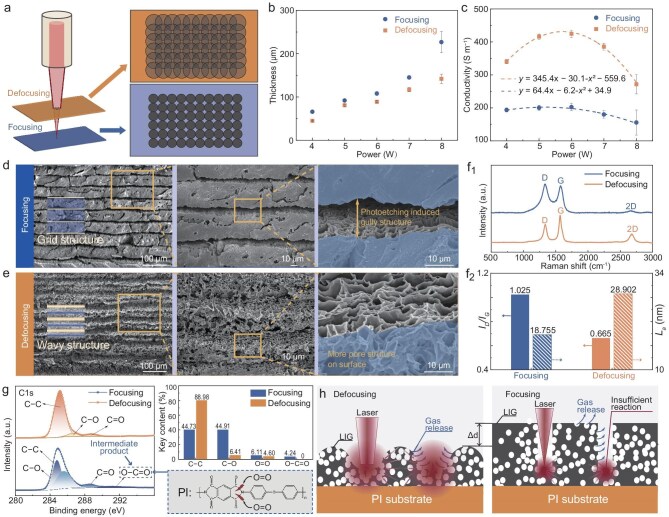
Comparison of laser-focused and laser-defocused prepared LIG. (a) Schematic illustration of the difference between laser-focused and laser-defocused scanning. (b) Thickness of F-LIG and DF-LIG fabricated under various laser powers. (c) Electrical conductivity of F-LIG and DF-LIG fabricated under various laser powers. (d and e) SEM images of F-LIG and DF-LIG surface at different magnification. (f) Raman spectra, *I_D_*/*I_G_*, and *L_a_* of F-LIG and DF-LIG. (g) XPS spectra and key contents of F-LIG and DF-LIG. (h) Schematic illustration of the mechanism in F-LIG and DF-LIG fabrication.

Compared to conventional heating technologies [27], laser processing exhibits distinct advantages, including higher energy density and faster heating rates. Notably, thermogravimetric (TG) analysis (Fig. S7) reveals that PI undergoes complete decomposition at ∼700°C with a heating rate of 5°C min^–1^ under atmospheric conditions. In contrast, the temperature required for PI carbonization and C-*sp^3^* to C-*sp^2^* transformation significantly exceeds 700°C in LIG fabrication. This is supposed to be the result of unique fast laser pulses, which lead to an instantaneous photothermal effect, enabling rapid surface heating and cooling (Fig. S7). Simultaneously, the laser pulse induces a photoetching effect, applying stress to the PI surface [28,29], which facilitates the rupture of chemical bonds within PI molecules. Figure 2a presents a schematic illustrating the difference between laser-focused and laser-defocused scanning on the PI surface. The irradiated area of the defocused laser is larger than that of the focused laser, leading to decreased power density and more repeated scanning of a region. Figure 2b shows that the thickness of LIG prepared by laser-focused scanning (F-LIG) and laser-defocused scanning (DF-LIG) increases nearly linearly with power, as more PI molecules react with increased power. The electrical conductivity of LIG initially increases and then decreases with increasing pulse power (Fig. 2c). The initial increase is attributed to higher induced energy, which promotes the transformation of amorphous carbon with poor conductivity into stacked graphene sheets with improved conductivity. However, beyond a certain threshold power, excessive energy leads to an increasingly rapid gas-release-rate, resulting in the formation of more internal voids and degradation of surface morphology [26,30], thereby negatively affecting the overall conductivity. Specifically, DF-LIG exhibits better conductivity than F-LIG, owing to fewer defects and a more continuous surface morphology. Moreover, a laser power of 3 W provides insufficient energy to form LIG, resulting in almost no change on the PI surface. A laser power of 8 W is of limited practical value, as the excessive energy results in substantial deformation of prepared film (Fig. S8) and causes significant measurement errors in both thickness and conductivity. In this work, subsequent EMI shielding–related tests will focus on samples prepared under laser powers of 4–7 W.

To investigate the impact of focused and defocused laser scanning on the LIG’s microstructure, scanning electron microscopy (SEM) characterization is adopted. DF-LIG and F-LIG exhibit obviously different surface morphologies, as the higher energy density in a focused-laser pulse facilitates the photoetching effect, thus leading to deep and narrow gullies on F-LIG (Figs 2d and S17). In contrast, a defocused-laser contributes to a broader range but weaker photoetching effect, resulting in a corrugated surface structure with shallow gullies and a more porous upper surface in DF-LIG (Figs 2e and S18). It can be demonstrated that the produced gases escape across the whole surface in DF-LIG, while they preferentially pass through pores on the ‘cliff face’ in F-LIG. The D peak, G peak, and 2D peak located at 1340 cm^−^^1^, 1567 cm^−^^1^, and 2677 cm^−^^1^ in the Raman spectra (Fig. 2f_1_) confirm the formation of LIG [31,32]. Both F-LIG and DF-LIG exhibit prominent D peaks, primarily due to: (1) the formation of an amorphous structure during the conversion of PI to LIG, followed by the growth into a stacked graphitic structure containing 5-, 6-, and 7-membered rings [33]. (2) The presence of impure elements N and O derived from PI molecules and oxygen in the air [34]. Significantly, DF-LIG exhibits a smaller *I_D_*/*I_G_* ratio of ∼0.67 compared to ∼1.03 in F-LIG, indicating fewer defects and high-degree C-*sp^3^* to C-*sp^2^* transformation, which is consistent with the higher electrical conductivity of DF-LIG (Fig. 2f_2_). Laser-defocused scanning promotes the photothermal effect, which enables temperature rise and enhanced graphitization. Furthermore, the crystalline size (*L_a_*) along the a-axis can be determined by the *I_D_*/*I_G_* ratio using the equation: *L_a_*  *=*  (2.4 × 10^−10^)   ×  λ_1_^4^ × (*I_G_*/*I_D_*) [35], where λ_1_ is 532 nm (wavelength of Raman laser). As is shown in Figs 2f_2_ and S19, compared to F-LIG, both the *L_a_* and *I_2__D_*/*I_G_* of DF-LIG are higher, suggesting enlargement of the crystalline domains and reduced stacks. Simultaneously, DF-LIG exhibits a noticeable narrowed and sharpened 2D band. These results imply that a defocused laser pulse is beneficial to the expansion of graphene layers, resulting in improved quality.

The decomposition of PI and subsequent formation of LIG involves bond breaking (e.g. –O– and C–N) and bond forming (e.g. C–C and C–O). Consequently, we conducted further analysis of the atomic binding state and element content in F-LIG and DF-LIG using X-ray photoelectron spectroscopy (XPS) (Figs. 2g and S20). The C–C bond content in DF-LIG (88.98%) is significantly higher than that in F-LIG (44.73%), while the content of C–O and C=O bonds is lower, indicating a higher degree of carbonization and graphitization in DF-LIG [36]. In PI molecules, the key bond energies of –O– and C–N are relatively low, causing them to preferentially dissociate under laser irradiation. Consequently, no C–N bonds were detected in the XPS results of F-LIG and DF-LIG. The increased C–O bonds in F-LIG may result from unreacted bonds in PI and the enhanced photoetching effect, which induces C dangling bonds that react with oxygen molecules in the air. Importantly, the O=C–O bond, as an intermediate product (Fig. S21), was detected in F-LIG, further indicating that DF-LIG underwent a more complete reaction resulting in improved electrical conductivity.

Based on the aforementioned experimental investigation, laser-focused/defocused-dependent formation of LIG is summarized (Fig. 2h): (1) when the laser is focused, energy is more concentrated with higher energy density, resulting in a more pronounced photoetching effect. Consequently, the PI surface rapidly forms a deep pit (steep cliff), where the photothermal effect causes heat to diffuse to surrounding areas, resulting in increased LIG thickness but insufficient crystalline domains. As a result, F-LIG exhibits more defects and lower electrical conductivity. (2) When the laser is defocused, enlargement of the laser-irradiated area improves energy distribution with lower energy density, resulting in a decreased photoetching effect. Meanwhile, the photothermal effect is enhanced which leads to increased growth of crystalline domains, resulting in fewer defects and better electrical conductivity.

### Electromagnetic modulation performance and mechanism of metasurfaces

**Figure 3.**
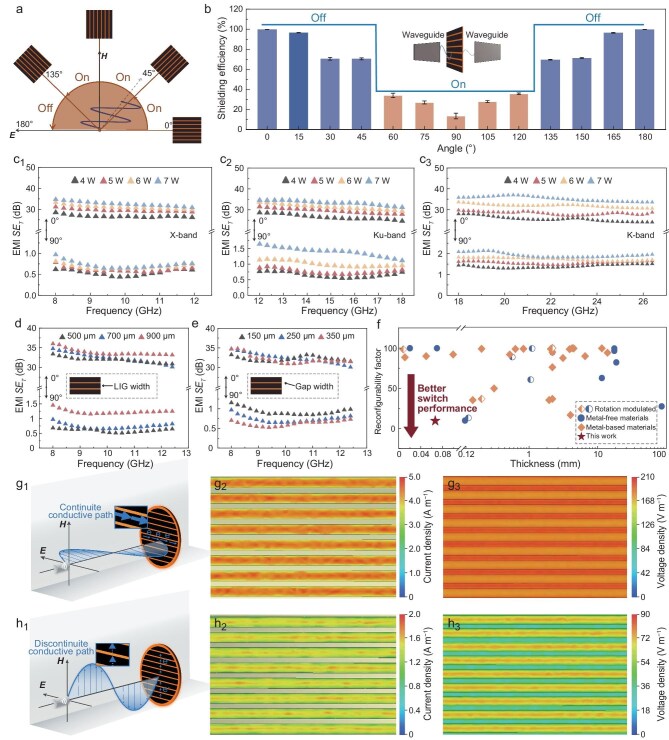
Performance and mechanism of the EMI shielding modulation. (a) Schematic showing the rotation process of the metasurface. (b) EMI shielding efficiency in various rotating angles at X-band. (c) EMI *SE_T_* of X-band, Ku-band and K-band. (d and e) EMI *SE_T_* at X-band with various LIG width and gap width. (f) Comparison of the reconfigurability factor (*r*) between reported works and this work. (g and h) Schematic illustration of the EMI shielding modulation performance, current density and voltage density simulated by CST software.

To date, thinner materials with higher conductivity are typically preferred due to their greater potential in miniaturized shielding systems [37–39]. Consequently, we have adopted DF-LIG for subsequent exploration of EMI shielding performance. As depicted in Fig. 3a and b, this metasurface shows continuous and dynamic regulation of electromagnetic waves. When the rotation angle ranges from 0° (***E***//LIG) to 45° and from 135° to 180°, the metasurface is in the Off-state, shielding >60% of the waves. Conversely, when the rotation angle ranges from 45° to 135°, the metasurface is in the On-state, transmitting >60% of the waves. Further insights into the coefficients of transmission (*T*), reflection (*R*), and absorption (*A*) are displayed in Fig. S22. With the angle increasing from 0° to 90° (***E***⊥LIG), *A* initially increases and then decreases under 8–26.5 GHz, while *R* generally shows a downward trend. These results reveal that the reflection of electromagnetic waves gradually reduce when the angle increases, which can be explained by the discontinuous conductive path and reduced secondary radiation (Note S9).

To explore the performance of this metasurface over a wide frequency band, the EMI SE was tested under 8–26.5 GHz (Fig. 3c_1_–c_3_). This metasurface exhibits an inverted response to incident waves, demonstrating impressive On/Off switchable tunability across a broad frequency range (8–26.5 GHz). Laser power plays a critical role in the total EMI shielding *SE* (*SE_T_*) in the On-state, with *SE_T_* exhibiting a positive correlation with the laser power. By integrating the preceding experimental results from Fig. 2c, it can be concluded that the modulation performance is collectively influenced by the electrical conductivity and thickness of the LIG, providing a fundamental basis for future optimization of its functionality. Notably, the minimum achievable *SE_T_* is only 0.441 dB at 9.85 GHz (laser power of 4 W), corresponding to a shielding efficiency of 9.66%. In contrast, the metasurface exhibits *SE_T_* of 26.6 dB (shielding efficiency of 99.998%) in the Off-state at this frequency. This achievement is critical as it not only shows remarkable Off-state performance, but also addresses the challenge of suboptimal On-state performance in reported works, where the shielding efficiency is too high with the majority exceeding 50% (Table S2). To assess behavior under realistic conditions, we conducted oblique-incidence tests which demonstrate that the LIG metasurface can basically maintain the On/Off switchable functionality over incident angles from 0° to 80° (Fig. S31).

Furthermore, a mechanistic study was adopted on absorption loss (*SE_A_*) and reflection loss (*SE_R_*) (Figs S23 and S24). *SE_T_* shows no obvious change with different frequency bands under the Off-state, but *SE_A_* and *SE_R_* show an opposite trend. With the frequency increase, *SE_R_*is reduced due to improved transmission performance and increased conductor loss of high frequency electromagnetic waves leads to enhanced reflection of incident waves; *SE_A_* is increased overall because the distance between waveguide ports is fixed while incident wavelength is decreased, contributing to a longer equivalent transmission path (Note S10). The results that the highest *SE_R_* at 6 W laser power under Ku-band and K-band is consistent with the higher electric conductivity tendency of LIG, originated from improved conductivity inducing poorer impedance matching, thus leading to enhanced reflection of electromagnetic waves (Note S11).

Further investigation of EMI *SE_T_* with varying LIG width (Fig. 3d) and gap width (Fig. 3e) revealed that a decrease in LIG width and an increase in gap width results in a lower *SE_T_*. Simultaneously, both widths show minimal impact on *SE_T_* in the Off-state with the average value being always higher at 30 dB (Figs S28 and S29). In Fig. 3f and Table S3, we present a comparison of thickness and reconfigurability factor (*r*) between this work and reported works. Critically, pursuing for higher EMI *SE* usually means utilization of thicker materials. Thickness as such should be of significant concern when considering application in future high-integrated electronics [40–43]. The factor *r* is used as a measure of the On/Off switchable performance and is calculated as follows:


\begin{eqnarray*}r\ = \ S{E}_{on\% }\ \cdot \ {f}_{on}\ + \ S{E}_{{\it off}{\%}}\ \cdot \ {f}_{\textit{off}},
\end{eqnarray*}

\begin{eqnarray*}{f}_{on}\ = \ 1\ -\ \frac{{S{E}_{\max \% \ }-\ 50\% }}{{S{E}_{\max \% }}},\end{eqnarray*}

\begin{eqnarray*}
{f}_{\textit{off}}\ = \ 1\ -\ \frac{{50\% \ -\ S{E}_{\min \% }}}{{S{E}_{\max \% }}},
\end{eqnarray*}

where *SE_on_*_%_, *SE_off_*_%_, *f_on_*, and *f_off_* represents the shielding efficiency in the On/Off state and corresponding factor, respectively. *SE*_max%_ and *SE*_min%_ represents the maximum and minimum shielding efficiency in reported works on EMI shielding dynamic regulation. The middle value is adjusted from reported 20 dB [44] to 50% for evaluating On/Off switchable shielding performance. Our LIG-based switch exhibits significant advantages, including reduced thickness and enhanced electromagnetic shielding modulation performance (a *r* value of 9.66), compared to reported metal-free and metal-based materials (Fig. 3f).

To explore the mechanism of modulated shielding performance, simulation of electromagnetically induced currents on the metasurface was performed using CST Microwave Studio (Fig. 3g_1_ and h_1_). The unit cell structure is simulated with periodic boundary conditions along the x and y axes, while Floquet port excitations are applied along the z direction (Fig. S30). When the direction of the electric field is parallel (***E***//LIG) and perpendicular (***E***⊥LIG) to the strip-arranged graphene, respectively, the incident electromagnetic wave induces a current in the conductive LIG, generating a secondary electromagnetic wave that opposes the incident wave. The simulated results for current and voltage densities in Fig. 3g_2_ and g_3_ (***E***//LIG) are significantly higher than those in Fig. 3h_2_ and h_3_ (***E***⊥LIG). The maximum current density is 5.19 A m^–1^ (***E***//LIG) and 2.78 A m^–1^ (***E***⊥LIG), and the maximum voltage density is 5860 V m^–1^ (***E***//LIG) and 1371 V m^–1^ (***E***⊥LIG). In the case of ***E***⊥LIG, the discontinuous conductive path limits the induced current density and voltage, leading to weaker secondary electromagnetic wave energy. Consequently, most of the incident waves are transmitted. Microscopically, the electrons in LIG oscillate back and forth in response to the change in the direction of the electric field, accompanied by secondary radiation. The wavelength of the incident waves (8–26.5 GHz) exceeds 1 cm, which is significantly larger than the gap between the strip-arranged LIG. When ***E***⊥LIG, the charged state of LIG changes only when the electric field direction alters, limiting the energy acquisition of electrons (Fig. 3h_2_ and h_3_) and preventing outward radiation. Consequently, the incident energy is primarily converted into transmitted energy. When ***E***//LIG, electrons move for extended periods with unchanged trajectories, thereby acquiring more energy. As a result, most of the incident energy is converted into electron kinetic energy (Fig. 3g_2_ and g_3_), and less energy is transmitted.

### Metasurface-based applications

**Figure 4.**
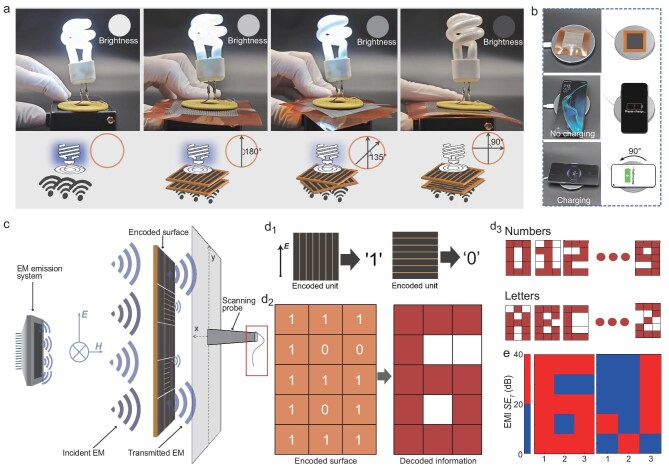
Application of the EMI shielding metasurface. (a) Light bulb brightness steerable phenomena controlled by two metasurfaces. (b) Wireless charging and no-charging controlled by the metasurface. (c) Schematic of electromagnetic near-field scanning test. (d_1_–d_3_) Schematic showing the application of information encoding. (d_1_) Definition of ‘0’ and ‘1’ for coding. (d_2_) Arrangement of coding units in number ‘6’ encoding. (d_3_) Schematic of encoding ability of all numbers and letters. (e) Electromagnetic near-field scanning test of the encoded surface.

To showcase the application prospects of the EMI shielding modulation, we designed a series of scenarios utilizing the dynamic tunability of the electromagnetic wave. As depicted in Fig. 4a, the brightness of a light bulb can be modulated by tuning the stacking angle between metasurfaces. When the two metasurfaces are stacked perpendicularly, the majority of waves are blocked, and the induced current is too small to light the bulb. With the angle altered from 90° to 180°, the brightness gradually increases originating from enhanced current caused by less shielded waves. These variations in visible light output demonstrate its feasibility in smart electronics. Furthermore, its On/Off switchable function in a wireless charging system is demonstrated (Fig. 4b). The switching between charging and non-charging states can be easily achieved via rotating the metasurface by 90°. This experiment showcases EMI shielding’s potential in the real-time control of wireless systems.

Significantly, we designed a novel paradigm for data electromagnetic encryption. Figure 4c illustrates the application and near-field scanning test process. Tests were performed in a microwave anechoic chamber (Fig. S32), where electromagnetic waves were emitted from the EM emission system, and a sliding-shaft scanning probe measured the field intensity behind each encoded unit. The unit with LIG//***E*** is defined as ‘1’, and the unit with LIG⊥is defined as ‘0’ (Fig. 4d_1_). As such, the encoded surface enables a distinct patterned electromagnetic response through various permutations and combinations of ‘0/1’. An encoded surface with 3 × 5 units was designed to encode all numbers (0–9) and letters (A–Z), where the arrangement of red cells enables visual patterning (Fig. 4d_2_ and d_3_). Figure 4e exhibits the near-field scanning results, which clearly show the preset encoded information of ‘6’ and ‘j’. In addition, this strategy enables effective discrimination of visually ambiguous characters (Fig. S33), including numerals vs. letters (e.g. ‘1’ vs. ‘I’ and ‘0’ vs. ‘O’) and uppercase vs. lowercase letters (e.g. ‘J’ vs. ‘j’). Moreover, a larger number of data points (such as color, transparency, depth, etc.) can be encoded in a single surface through the different orientation of units from 0° to 90°, thus enabling, for instance, the encoding of a colored QR code storing substantial amounts of information. Especially in the process of encoding and decoding, the following factors jointly determine the final results: (1) the wavelength and direction of incident waves, (2) the arrangement design of the encoded surface, (3) the distance between the surface and scanning probe, (4) the corresponding information detected from the electromagnetic strength. Therefore, a one-to-one mapping of incident wave-encoded surface-decoded information can be established, thereby enabling the as-prepared metasurface to be applied in electromagnetic encryption and decryption of critical information.

## CONCLUSION

This work presents a smart LIG-based metasurface designed for exceptional dynamic modulation of electromagnetic waves. Utilizing laser-induced ultrafast kinetics, this metasurface achieves rapid preparation, open-air processability, and micron-level thickness. Furthermore, laser-defocused, as compared to laser-focused, scanning results in a more complete reaction and improved electrical conductivity of LIG. Notably, this smart metasurface exhibits a superior shielding efficiency modulation range of 9.66%–99.78% and a high reconfigurability factor of 19.31. The superior modulation capability is attributed to the fact that stronger electron oscillation leads to improved secondary radiation in the opposite direction to the incident wave, when ***E***//LIG (compared to ***E***$ \bot $LIG); as a result, the incident wave is effectively shielded. Finally, we demonstrate the metasurface’s significant capability for information encoding and even encryption, highlighting broad applicability in commercial multifunctional and military electronic systems.

## METHODS

### Materials

Polyimide (PI) film with a thickness of 150 μm (Zhuzhou Times New Materials Technology Co., Ltd, China) was directly subjected to irradiation by an infrared laser platform (ProtoLaser S, LPKF, Germany) in ambient conditions to fabricate LIG patterns. The laser source generates infrared radiation of 1064 nm wavelength. Before laser irradiation treatment, PI films were cleaned with ethanol to remove residues from factory production and cut into appropriate sizes (10 cm long and 2 cm wide). The pulse frequency of the laser is 200 kHz and the scanning speed is 15 mm/s. In order to investigate the structure and properties of LIG in focusing and defocusing laser mode processing, the focus height was set to 150 μm (on the surface of PI film) and 1500 μm (above the surface of PI film).

### Characterization

The surface morphology of the sample was characterized using a scanning electron microscope (JSM-7610F Plus, JEOL, Japan). Raman spectra were characterized with a Raman spectrometer (dxr3, Thermo Fisher Scientific, USA). Square resistance and conductivity measurements were taken of LIG prepared with different laser powers using a 4-Point Probes Resistivity Measurement System (RTS-8, four Probes Tech, China). A hyper-depth of field three-dimensional microscopic system (VHX-600E, Keyence, Norway) was adopted to capture high-definition images of the surface topography for measuring the minimum line width and inter-line gap in Figs 1B, S3, and S4. XPS data were collected with a Scanning X-ray Microprobe (ESCALAB 250Xi, Thermo Fisher Scientific, USA). The dynamic degradation studies for the samples were carried out in air and N_2_ atmosphere in a thermal analyzer (STA 449 F3 Jupiter, Netzsch, Germany). PI were heated from ambient to 1673 K at a rate of 5 K/min. Electromagnetic interference shielding measurements of the LIG metasurface were carried out in a rectangular waveguide using a vector network analyzer (N5225A, Keysight, 8–26.5 GHz). Frequency range measurements of 8.0–12.4 GHz (X-band), 12.4–18.0 GHz (Ku-band), and 18.0–26.5 GHz (K-band) are adopted in a corresponding type of waveguide. Based on the measured results, the coefficients of reflection (*R*), transmission (*T*), and absorption (*A*) can be determined by *R *= ${| {{{{S}}}_{{\textit{11}}}} |}^{\mathrm{2}}$, *T * = $| {{{{S}}}_{{\textit{21}}}} |$^2^, and *A* = 1 − *R* − *T*. The reflection loss (*SE_R_*), absorption loss (*SE_A_*), and total shielding effectiveness (*SE_T_*) can be determined as: *SE_R_ = *–10log(1 − *R*), *SE_A_ = *–10lg[*T*/(1 – *R*)], *SE_T_ = SE_R_ + SE_A_*. The shielding efficiency can be calculated as: [100 – 1/10^(*SE_T_*/10)] ${\mathrm{ \times }}$ 100%. The near-field shielding effectiveness performance of the LIG metasurface was carried out by a measurement system in an anechoic chamber. In this system, a scanning probe was employed to capture a near field signal leakage (magnetic field signal, *H*) from the covered shielding materials. One end of the micro-strip antenna and the scanning probe was connected to port 1 and port 2 of the VNA, respectively.

## Supplementary Material

nwaf395_Supplemental_File
