# Supplementary material for: Superior microwave shielding modulation based on rapidly prepared graphene metasurface
Source: Natl Sci Rev. 2025 Sep 17;12(11):nwaf395. doi: 10.1093/nsr/nwaf395 (PMC12560093; doi:10.1093/nsr/nwaf395)
Supplement: nwaf395_Supplemental_File [file nwaf395_supplemental_file.pdf]

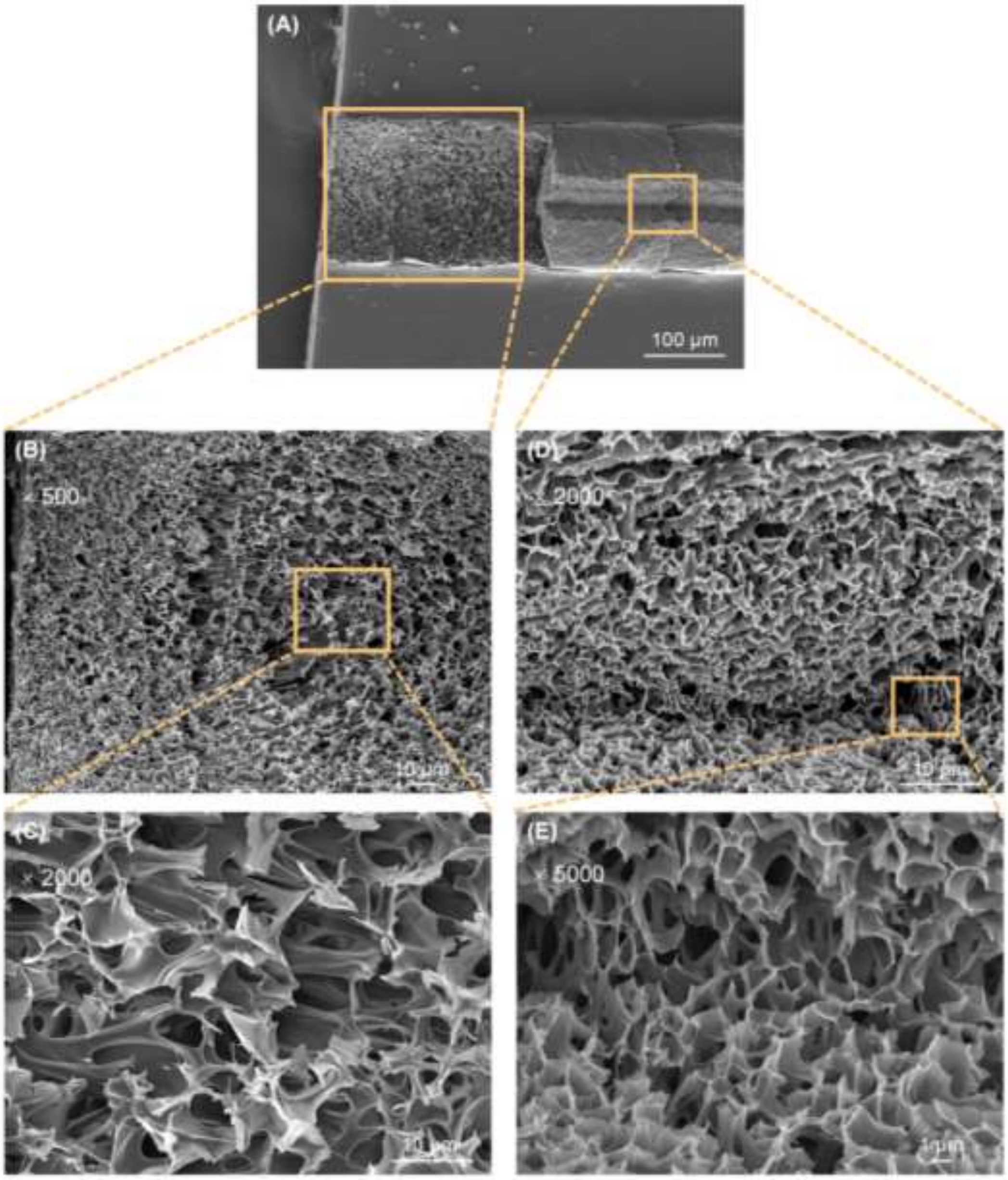

Figure S1. SEM images of laser-induced graphene (LIG) at various magnifications.

Note S1: In our ongoing research, we have observed that the distance between the line-scanning route affects the structure and properties of LIG, as the interactions between multiple scans lead to secondary reactions, photoetching of the reacted areas, and other related phenomena. To effectively demonstrate the characteristics of laser-induced technology and LIG's structure, a one-line scanning prepared LIG with

typical structure was selected for SEM characterization. The impact of laser on the surface of the PI film can be mainly devided into photoetching and photothermal effect. The photoetching effect will lead to the ablation of PI surface thus forming the groove structure (the right box in **Figure S1A**). The photothermal effect will lead to the ultrafast increasing of temperature and promote the conversion of PI molecules to graphene. In order to clearly observe the internal structure, we carefully peeled off the upper layer of LIG (the left box in **Figure S1A**). A significant amount of gas is released during this process, contributing to the formation of a relatively ordered pore structure (**Figure S1B–D)**. Interestingly, compared with the internal and groove part, the surface of LIG is relatively smooth with few pores. We speculate that the photoetched groove structure provides paths for gas release, as the similar pore structure observed in **Figure S1B–E.**

The C–N bonds with relatively low bond energy (3.04 eV) are preferentially decomposed, leading to the formation of micro-cracks and changes of surface morphology[1-3]. This, in turn, alters the optical properties of PI films and enhances their infrared absorption[4, 5], thereby promoting heat accumulation in PI films. At 550 ℃, PI film releases CO_2, N_2 and CO through the dissociation of C=O, C–N, and C–O bonds[6, 7]. Carbonization initiates above 800 ℃, resulting in the decomposition of complex PI chains into simpler carbon structures. This process releases volatile components, leaving behind a disordered, carbon-rich residue. When temperature exceeds 1700 ℃, amorphous carbon (sp^3) transforms into stacked ordered graphene sheets (sp^2)[8-10].

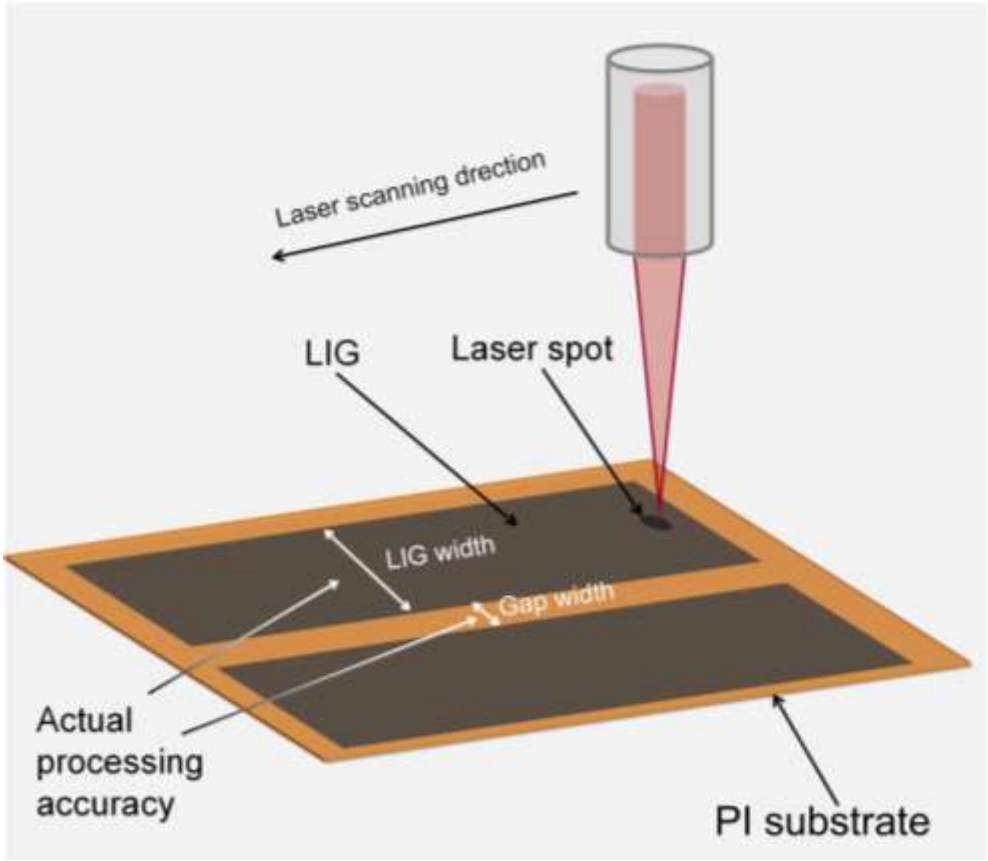

Figure S2. Schematic diagram of laser scanning process.

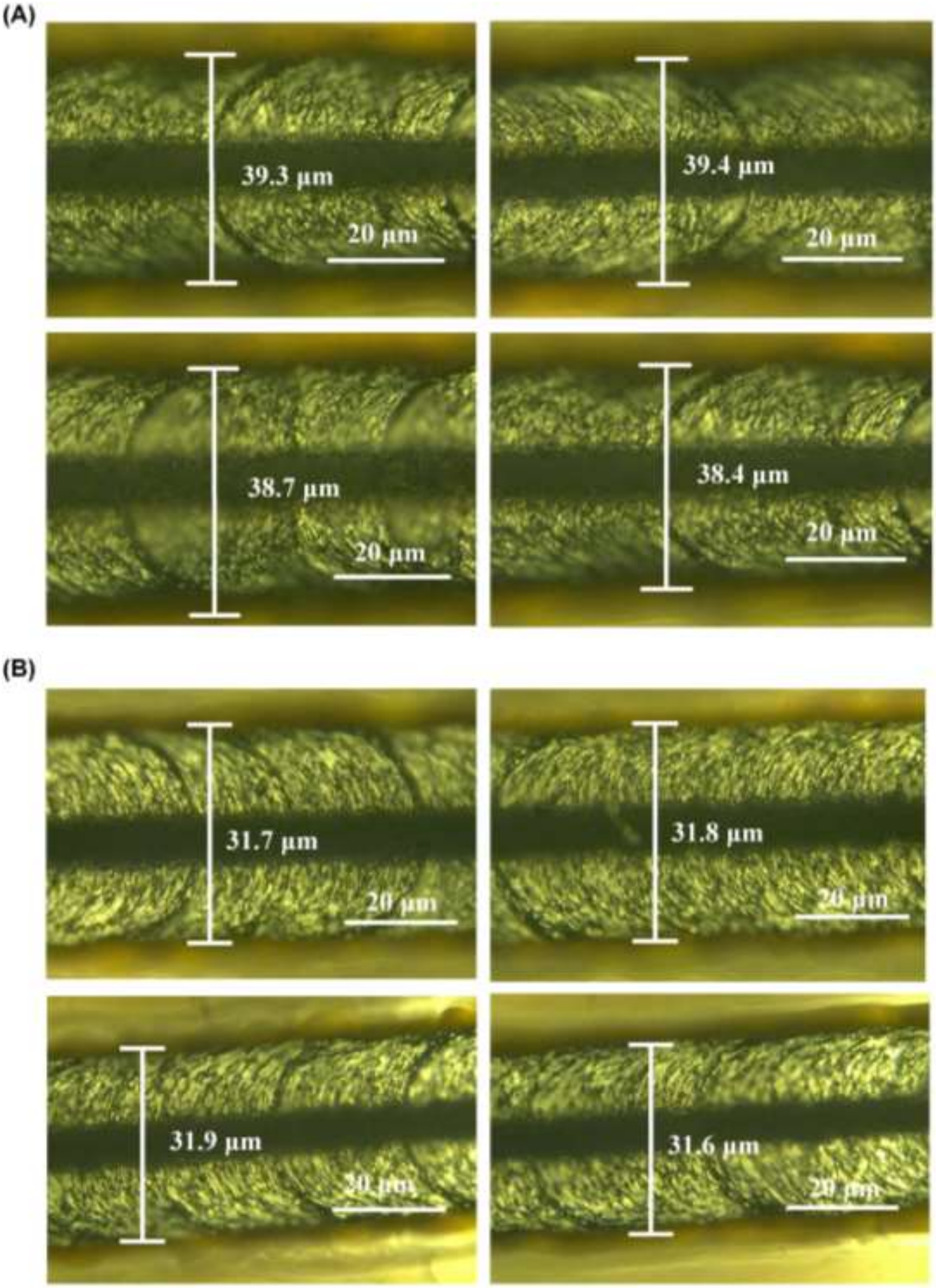

Figure S3. Optical image of the LIG prepared by single-line laser scanning under the condition of defocusing (A) and focusing (B).

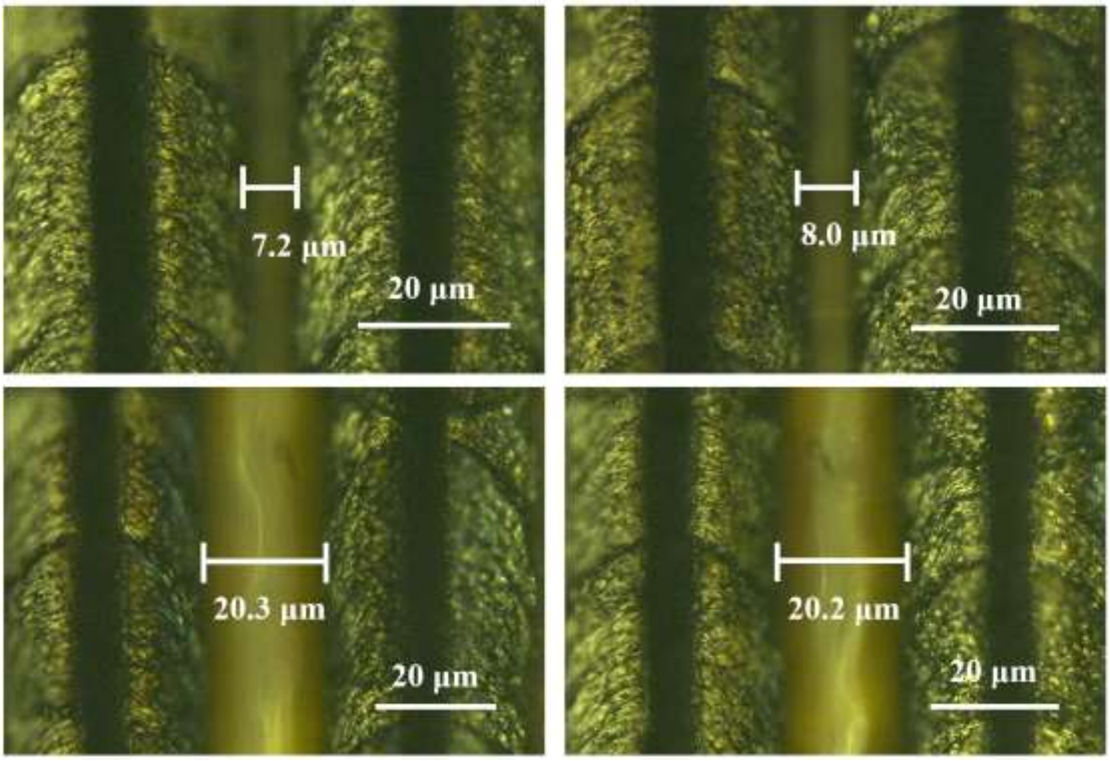

Figure S4. Optical image of the LIG prepared by two-lines laser scanning with various gap width.

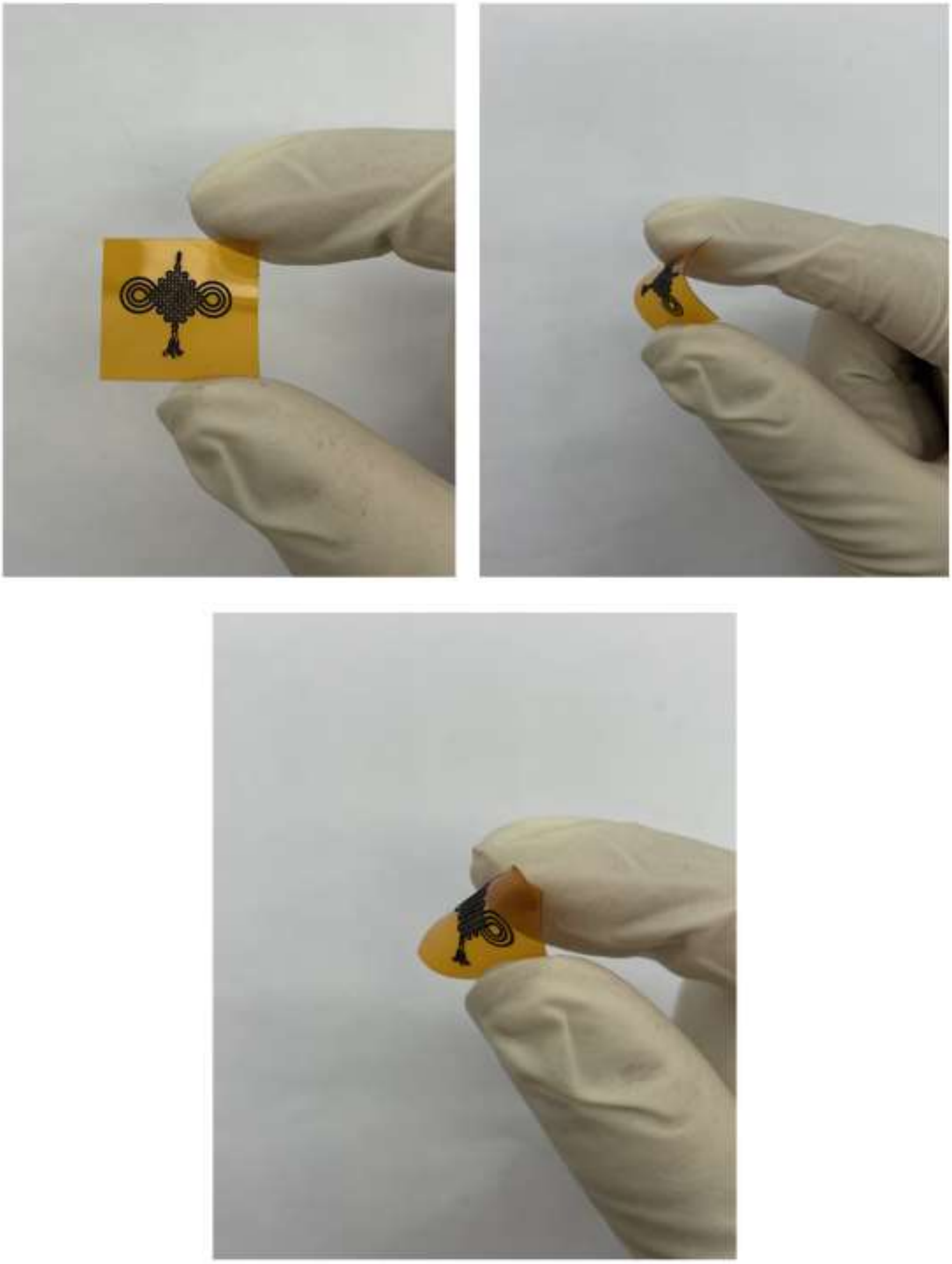

Figure S5. Photographs of the Chinese-knot-shaped LIG.

Table S1. Comparison of typical parameters in laser-induced graphene technology among reported works and this work.

Num.	Laser wavelength (µm)	Laser spot diameter (focusing)	Actual processing accuracy (µm)	Ref.
1	10.6	120	Not mentioned	[6]
2	0.355	26.5	Not mentioned	[11]
3	10.6	76	Not mentioned	[12]
4	10.6	200	Not mentioned	[13]
5	10.6	120	Not mentioned	[14]
6	1.064	430	Not mentioned	[15]
7	0.410	200	Not mentioned	[16]
8	10.6	800	Not mentioned	[17]
9	10.6	125	Not mentioned	[18]
10	9.3	> 61.8	Not mentioned	[19]
11	10.6	150	Not mentioned	[20]
12	1.064	25	31.75[1] (line)<br>7.6[2] (gap)	This work

[1]: The LIG width fabricated under one-line laser scanning on PI. The value is obtained by averaging the results from multiple samples processed under the same laser scanning parameters.
[2]: The gap width between the LIG fabricated under two independent one-line laser scanning on PI. The value is obtained by averaging the results from multiple samples processed under the same laser scanning parameters.

Note S2: Actual processing accuracy rather than theoretical accuracy dictates the precision of the patterning design, especially for laser-induced reaction. As displayed in **Figure S2**, the actual processing LIG width is larger than that of laser spot diameter, which means the actual processing accuracy cannot be equated with the processing accuracy indicated in the laser equipment manual. This is because that the photothermal effect induced by the high-energy pulse will extend beyond the irradiated area, leading to reduced patterning accuracy. Therefore, investigating the actual thermal influence range of the laser's photothermal effect is essential. In this work, we focus on both LIG width and gap width as they jointly determine whether the subsequent patterning of the grid-like metasurface can be accurately achieved. To

ensure experimental reproducibility, multiple repetitions were performed under the same conditions (**Figure S3**). Under laser-defocused scanning, the minimum width of LIG width were determined to be 38.4–39.4 μm with the average value of 38.95 μm and the error range of ~1.41%. Under laser-focused scanning, the minimum width of LIG width were determined to be 31.6–31.9 μm with the average value of 31.75 μm and the error range of ~0.47%. The above results demonstrate the consistency of the multiple experiments. Based on above exploration on LIG width, the accurate control of gap width depends more on the mechanical displacement accuracy of related components in laser processing system. Therefore, we set the gap widths to 7.5 μm and 20 μm to investigate the discrepancy between the actual and set results. The error ranges were determined to be 6.67% and 1.5%, respectively (**Figure S4**), demonstrating that a larger set gap width results in higher accuracy in actual processing. In addition, we then give a comparison of typical parameters among reported works and this work (**Table S1**). No attention has been paid to the actual processing accuracy in LIG technology to the best of our knowledge. Thus, our efforts on this issue would provide a solid foundation for ultrafast preparation of patterned surface based on LIG technology.

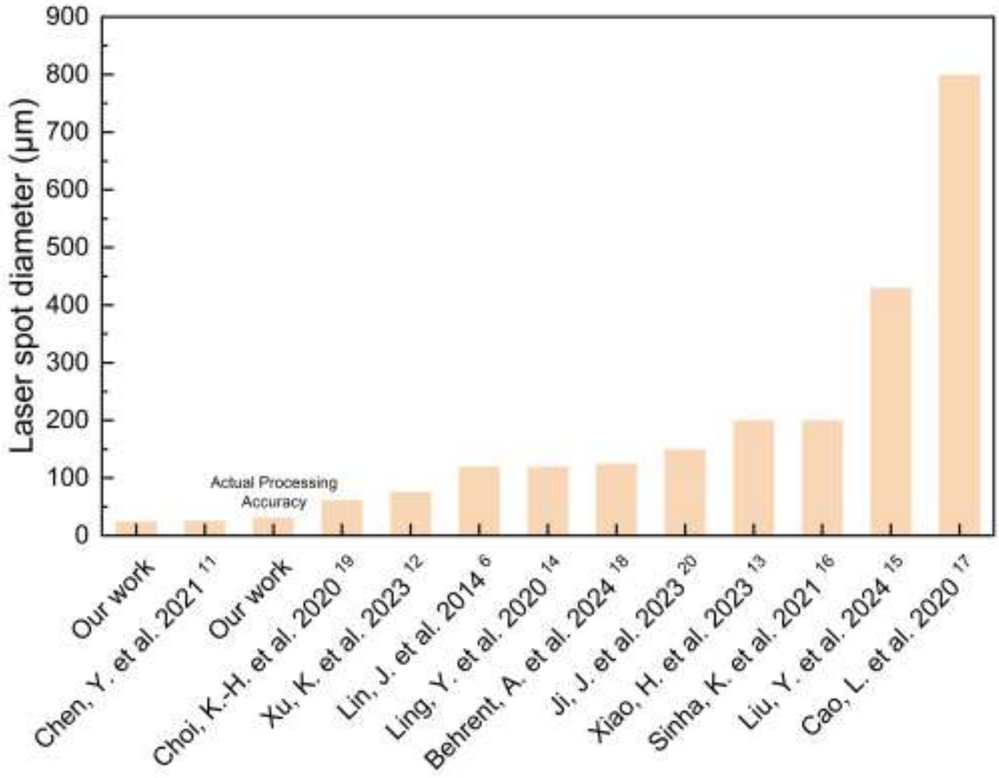

Figure S6. Comparison of laser spot diameter in reported works and this work.

Table S2. Statistical table of dynamic EMI shielding performance in reported work and this work.

No.	Type of tunability	Material type	Materials	Frequency (GHz)	Off-state *SE* (dB)	Off-state $SE_{\%}$[1] (%)	On-state *SE* (dB)	On-state $SE_{\%}$[1] (%)	Synthesis duration (h) Total	Synthesis duration (h) Detailed	Size (mm)	Thick-ness (mm)	Weight (g)	Ref.
1	Compression	Metal-free	Wood-derived carbon/XC-72 NP aerogel	8–12.4	27.6	99.826	1.4	27.556	44	Hydrothermally treating wood with sodium hydroxide and sodium sulfite: 24 h<br>Hydrothermally treating wood with hydrogen peroxide: 4 h<br>Acidifying Vulcan carbon nanoparticles: 8 h<br>Stabilizing wood/Vulcan carbon sponge: 4 h<br>Carbonizing wood-derived carbon aerogel: 4 h	22.8 × 10.0	6.9	/	[21]
2			WF/CNTs composite foam	8–12.4	40.1	99.990	7.5	82.217	86.3	Stirring wheat flour and carbon nanotube: 10 min<br>Freezing wheat flour/carbon nanotube gel: 12 h<br>Freeze-drying wheat flour foam: 48 h<br>Treating foam with 3-(methacryloxypropyl) trimethoxysilane: 2 h<br>Freeze-drying hydrophobic foam: 24 h<br>Hot-pressing foam into film: 10 min	16 ×16	16	/	[22]
3			PU/CNTs/TPI	8–12.4	62	99.999	4.3	62.846	6	Ultrasonically dispersing carbon nanotubes: 2 h<br>Stirring trans-1,4-polyisoprene and dicumyl peroxide: 4 h<br>Cross-linking trans-1,4-polyisoprene: 15 min	/	/	/	[23]
4			PU/RGO foam-5%	8–12.4	22.3	99.411	14.7	96.612	4	GO coating: 2 h<br>PUG reducing: 2 h	30 × 20	15	/	[24]

5		PU/RGO foam-10%	8–12.4	39.4	99.989	23.4	99.543	4		30 × 20	15	/	[24]
6	Metal-based	TPI-MXene/ carbon foam	8–12.4	45	99.997	25	99.684	158.8	Etching titanium aluminum carbide: 48 h<br>Stirring cetyltrimethylammonium bromide with titanium carbide: 2 h<br>Stirring trans-1,4-polyisoprene and titanium carbide: 4 h<br>Drying balsa wood: 12 h<br>Treating wood with sodium hydroxide and sodium sulfite: 7 h<br>Treating wood with hydrogen peroxide: 24 h<br>Freeze-drying wood foam: 48 h<br>Carbonizing wood foam: 1 h<br>Impregnating carbon foam in solution: 30 min<br>Vacuum-drying carbon foam: 12 h<br>Cross-linking trans-1,4-polyisoprene: 15 min	/	/	/	[25]
7		EVA@PPy@ Ag foam	8–12.4	61.6	99.999	6.23	76.177	> 10.9	Stirring ethylene-vinyl acetate in cyclohexane: 1 h<br>Drying ethylene-vinyl acetate mixture: 4 h<br>Hot-pressing ethylene-vinyl acetate: 30 min<br>Immersing foam in iron chloride: 10 min<br>Vapor phase polymerization of pyrrole: 0.25–2 h<br>Soaking foam in silver nitrate: 3 h<br>Electroless silver plating: 2 h	15 × 15	5	/	[26]
8		Melamine foam@MXene/	8–12.4	30.5	99.911	12.4	94.246	60	Modifying melamine foam with polydopamine: 12 h<br>Freeze-drying composite: 48 h	30 × 30	30	/	[27]

			Ag nanowire sponges/PEG											
9			Melamine foam@Ag/PDA/ CNT/ waterborne polyurethane	8–12.4	40.1	99.990	25	99.684	66.2	Modifying melamine foam with polydopamine: 17 h<br>Stirring carbon nanotubes and waterborne polyurethane: 1 h<br>Freezing mixture: 10 min<br>Freeze-drying foam: 48 h	20 × 20	4	/	[28]
10			Identical patterned anisotropic magnetic liquid metal/PDMS	8–12.4	45	99.997	7	80.047	6.9	Preparing magnetic liquid metal: 30 min<br>Drying magnetic liquid metal: 2 h<br>Stirring and curing (first step): 10 min + 30 min<br>Stirring and curing (second step): 15 min + 3.5 h	/	/	/	[29]
11	Stretching	Metal-free	CNT/TPU	8–12.4	34.57	99.965	12.8	94.752	1.2	Ball-milling TPU and carbon nanotubes: 1 h<br>Microwave sintering: 45 s<br>Compression molding: 10 min	40 × 10	2	/	[30]
12			PEDOT:PSS/ waterborne PU	8–12.4	62	99.999	< 62	99.999	12.5	Stirring PEDOT:PSS and waterborne polyurethane mixtures: 0.5 h<br>Drying composite films: 12 h	20 × 2	0.15	0.054	[31]
13		Metal-based	pre-linked Ni chains elastomer	8–12.4	23	99.499	3	49.881	> 13.2	Modifying nickel powders: 7 h<br>Stirring mixture: 3–15 min<br>Vacuum degassing: 10 min<br>Curing elastomer: 6 h	40 × 20	2	/	[32]
14			Fe/liquid metal/PDMS	8–12.4	80.7	99.999	20.6	99.129	16	Purification and drying of MLM: 12 h (overnight)<br>Prepearation of AMLM film: 4 h	/	/	/	[33]
15			3D liquid metal	2.65–5.95,	81.6	99.999	41.5	99.993	/	/	/	/	/	[34]

			network	8.2–40										
16			Liquid metal elastomer composite (BiInSn)	8–12.4	11	92.057	0.8	16.824		Mixing metals to prepare BiInSn: 4 h<br>Stirring BiInSn and nickel into Ecoflex: 20–30 min<br>Mixing parts A and B of Ecoflex: 3–5 min<br>Curing liquid metal foamed elastomer composite: 6 h	80 × 60	3.6	/	[35]
17			Liquid metal elastomer composite (Ga)	8–12.4	28	99.842	11	92.057	> 10.4					[35]
18			Ag NPs/SEBS	8–12.4	42.00	99.994	28.00	99.842	/	/	20 × 8	3	/	[36]
19			Liquid metal GaIn24.5/Ni	8–12.4	70	About 100	10	90	> 1.2	Preparing EGaIn alloy:1 h<br>Stirring nickel and EGaIn: 10–15 min	/	/	/	[37]
20	Temperature	Metal-based	VO_2/CNF	8–12.4	52.8	99.999	19.7	98.928	28.1	Ball-milling vanadium dioxide and cellulose nanofiber: 5 min<br>Freezing vanadium dioxide/cellulose nanofiber gel: 4 h<br>Freeze-drying aerogel: 24 h<br>Compressing aerogel into film: 1 min	/	/	/	[38]
21		Metal-free	core−shell structural PNIPAM@ p-PDA biomicrospheres	8–12.4	57	99.999	28	99.842	24.5	Stirring PNIPAM with dopamine: 12 h<br>Stirring PNIPAM@polydopamine with MXene: 12 h<br>Drying to form yolk-shell structure: 30 min	40 × 20	0.12	/	[39]
22	Humidity	Metal-based	VO_2/EPM composites	8–12.4	27.9	99.838	18.73	98.660	0.5	Heat treatment: 0.5 h	/	/	/	[40]

		foam										
23		VO_2/poly(vinylidene fluoride-cohexafluoropropylene)	8–12.4	18.4	98.555	1.9	35.435	69.2	First hydrothermal synthesis of vanadium dioxide nanowires: 24 h<br>Second hydrothermal synthesis of vanadium dioxide nanowires: 24 h<br>Annealing vanadium dioxide nanowires: 2 h<br>Stirring polyvinylidene fluoride-co-hexafluoropropylene solution: 2 h<br>Stirring vanadium dioxide nanowires in solution: 1 h<br>Stirring mixture of components: 4 h<br>Vacuum-drying composite film: 12 h<br>Hot-pressing composite film: 10 min	/	/	/	[41]
24		RGO/VO_2-300 ℃	8–18	14.7	96.612	9.1	87.697	63	Hydrothermal synthesis of vanadium dioxide: 18 h<br>Stirring vanadium dioxide: 6 h	/	/	/	[42]
25		RGO/VO_2	8–12.4	37	99.980	10	90		Vacuum-drying vanadium dioxide derivatives: 2 h<br>Stirring vanadium dioxide derivatives with graphene oxide: 1 h<br>Solvothermal reaction for hydrogel: 12 h<br>Solvent exchange for hydrogel: 24 h	/	/	/	[42]
26		$Ti_3C_2T_x$-WVO_2	8–12.4	42.8	99.995	28.9	99.871	73.4	Etching titanium aluminum carbide: 48 h<br>Centrifuging titanium carbide: 6 min<br>Centrifuging vanadium oxyhydroxide precursor: 10 min<br>Stirring ammonium para-tungstate solution: 1 h<br>Hydrothermal reaction for tungsten doped	/	/	/	[43]

										vanadium dioxide: 24 h<br>Centrifuging tungsten doped vanadium dioxide: 10 min				
27	Electrochemical potential	Metal-free	Pyrolytic graphite-wet RGO/CNTs/PP non-woven spacer-pyrolytic graphite	8–12.4	7	80.047	2	36.904	23	Heating graphene oxide/carbon nanotube/non-woven mixture: 11 h<br>Purification and drying of graphene oxide/carbon nanotube/non-woven spacers: 12 h (overnight)	/	2	/	[44]
28		Metal-based	MXene film ($Ti_3C_2T_x$ electrode in 1 M H_2SO_4)/PET	8–12.4	12.45	94.311	32.98	99.949	44	Etching vanadium aluminum carbide: 18 h<br>Delaminating MXenes: 26 h	/	/	/	[45]
29	Rotation	Metal-free	PVB/Ni-graphene/short-cut CF composite films	8–12.4	31.8	99.934	20.2	99.045	9	Stirring suspension: 6 h<br>Removing bubbles: 3 h	/	0.2	/	[46]
30			CF reinforced polymer	5–15	40	99.990	10	90	1.5	Fabrication of unidirectional carbon fiber reinforced polymer composites: 1.5 h	250 × 250	0.55	/	[47]
31			CF hybrid fabrics		4.50	64.519	4.37	63.441	/	/	/	/	/	[48]
32			RGO@DPC	12–18	25	99.684	17	98.005	55	Preparation of DPCs and RGO@DPCs: 34 h<br>Formation of gradient RGO@DPCs: 21 h	/	1.5	/	[49]
33			CNT/NFC	12–18	72	99.999	33.5	99.955	14	Pretreating substrates with sodium hydroxide: 2 h<br>Drying pretreated substrates: 12 h	/	/	/	[50]
34			Co based AWs	12–18	24.6	99.653	0.6	12.904	> 12	Electrophoretic depositing graphene oxide: 40 s<br>Hydrothermal reducing graphene oxide: 12 h	/	/	/	[51]

35		MXene film (V_2CT_x electrode in 1 M H_2SO_4)/PET	12–18	25.2	99.929	31.5	99.698	44	Preparation of MXene preparation: 18 h<br>Preparation of electromagnetic shielding devices: 26 h	/	/	/	[45]
36	Metal-based	MXene/CNF aerogel	8–12.4	42	99.994	13	94.988	142	Ball-milling MXene precursors: 18 h<br>Annealing MXene precursors: 4 h<br>Etching titanium aluminum carbide: 24 h<br>Etching vanadium aluminum carbide: 48 h<br>Delaminating vanadium and niobium carbide: 48 h	120 × 60	2	0.0576	[52]
37		MXene@wood	8–12.4	57.6	99.999	27.4	99.818	38	Etching titanium aluminum carbide: 24 h<br>Drying MXene-coated wood: 2 h (air) + 12 h (60 °C)	120 × 60	0.0005	0.050	[53]
38		MXene@polydopamine	8–12.4	16.2	97.601	0.29	6.459	68	Preparation of $Ti_3C_2T_x$ MXene: 44 h<br>Preparation of p-MXene Nanosheets: 24 h	/	0.018	0.0082	[54]
39	Metal-free	LIG metasurface	8–12	25.35	99.710	0.54	11.774	0.83	Single-step	50 × 50	0.0668	0.88	This work
			12–18	26.09	99.754	0.66	14.099						
			18–26.5	25.59	99.724	1.40	27.525						
			9.85	26.88	99.783	0.44	9.655						

[1]: $$\text{Shielding efficiency} = \left(1 - 10^{-\frac{EMISE}{10}}\right) \times 100\%$$

Nanoparticles (NP), wheat flour (WF), carbon nanotube (CNT), polyurethane (PU), trans-1,4-polyisoprene (TPI), reduced graphene oxide (RGO), ethylene-vinyl acetate copolymer (EVA), polyvinyl butyral (PVB), polypyrrole (PPy), poly(ethyleneglycol) (PEG), poly-dopamine (PDA), polydimethylsiloxane (PDMS), 3 dimension (3D), styrene-(ethylenebutylene)-styrene (SEBS), poly(N-isopropylacrylamide)@porous polydopamine (PNIPAM@p-PDA), expanded polymer microsphere (EPM), poly(3,4-ethylenedioxythiophene)/poly(styrenesulfonate) (PEDOT:PSS), vanadium

dioxide (VO_2), directional porous carbon (DPC), amorphous wires (AW), polyprolene (PP), poly(ethylene terephthalate) (PET), carbon fiber (CF), nano-brillated cellulose (NFC), cellulose nanofibrils (CNF). Notably, synthesis times are reported as the sum of individual process durations, excluding inter-step intervals. While various factors influence synthesis duration, we present these data as a qualitative reference, underscoring the urgency of developing practical, industrially relevant synthesis methods for electromagnetic functional thin films.

Note S3: Traditional EMI shielding materials prioritize achieving higher shielding values, with the dB value commonly used as the metric for measuring EMI shielding performance. For instance, when shielding 99.9% and 99.99% of electromagnetic waves, the shielding values calculated are 30 dB and 40 dB, respectively, effectively illustrating subtle differences in EMI SE values in practical applications. However, this conversion fails to accurately reflect the actual scenario at low dB values, particularly when the EMI shielding switch is in the “On” state. For instance, when the EMI shielding performance is below approximately 10 dB, every 1 dB change results in a sharp increase in the actual EMI shielding efficiency, while 2-3 dB change corresponds to a 37%-50% difference in actual EMI shielding efficiency. For an EMI shielding switch, it is more appropriate to use the actual EMI shielding efficiency (measured in %) as the performance metric.

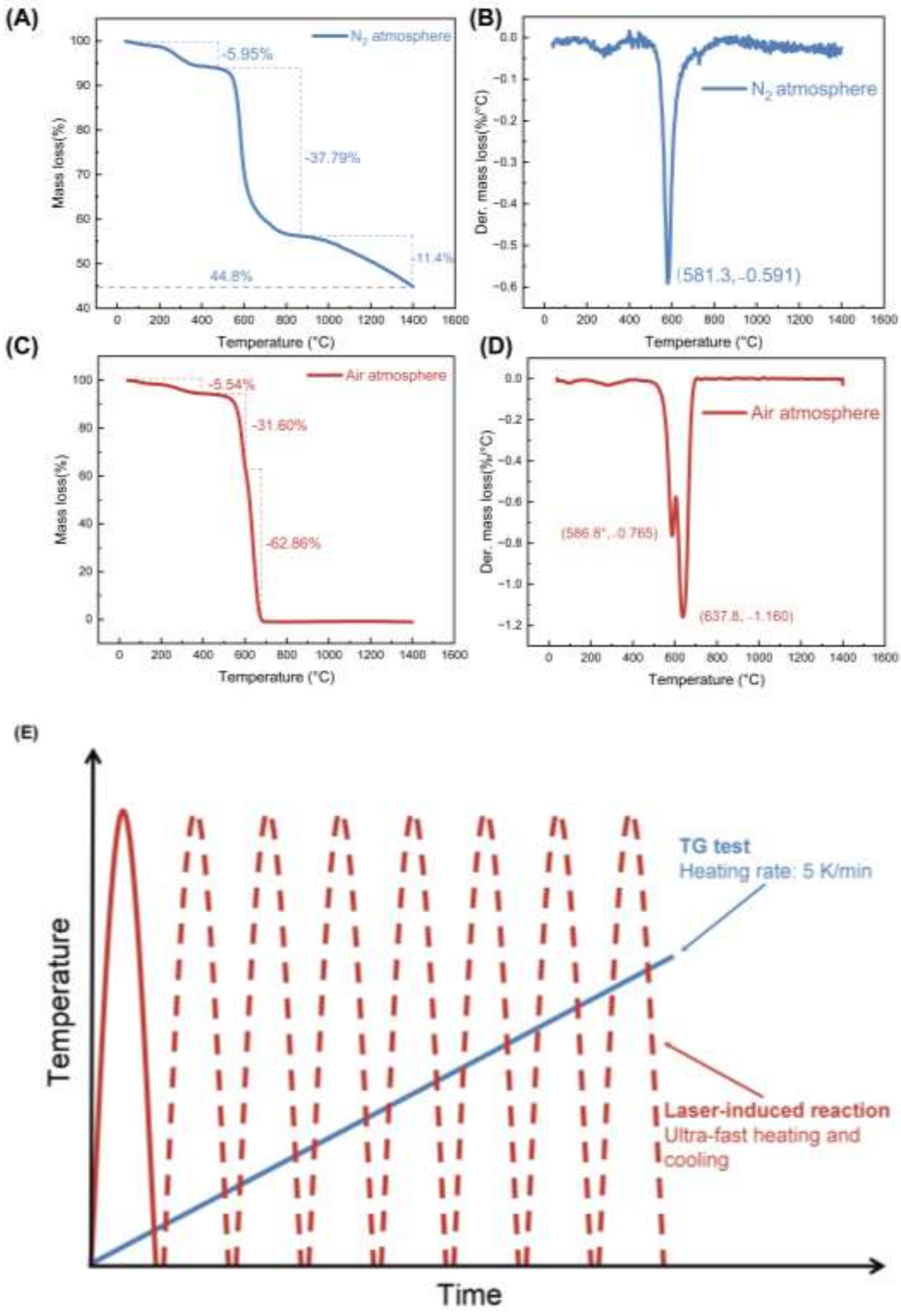

Figure S7. (A–D) Thermogravimetric analysis of PI film substrate in N_2 and air atmosphere. (E) Schematic diagram of the ultra-fast heating in laser-induced process and slow heating in TG test.

Note S4: To date, to the best of our knowledge, there has been no systematic investigations on the reaction mechanism of LIG technology. The invention of this technology was relatively late (in 2016) and laser-induced physical and chemical reaction is highly complex. Compared with laser processing on metals, such as wielding, engraving, etc., the researches on LIG technology mainly focus on the electronic devices design and construction, with the clear and comprehensive reaction process uncovered. Although the focus of this work is not the mechanism, we conducted TG test on PI film to contrast the difference between LIG technology's ultrafast kinetics and traditional heating process (**Figure S7**). The thermogravimetric (TG) analysis reveals that PI undergoes complete decomposition at approximately 700 ℃ with a heating rate of 5 ℃/min under atmospheric conditions. In contrast, the temperature required for PI carbonization and C-sp^3 to C-sp^2 transformation significantly exceeds 700 ℃ in LIG fabrication. This is supposed to be the results of unique fast laser pulses, which lead to an instantaneous photo-thermal effect, enabling rapid surface heating and cooling.

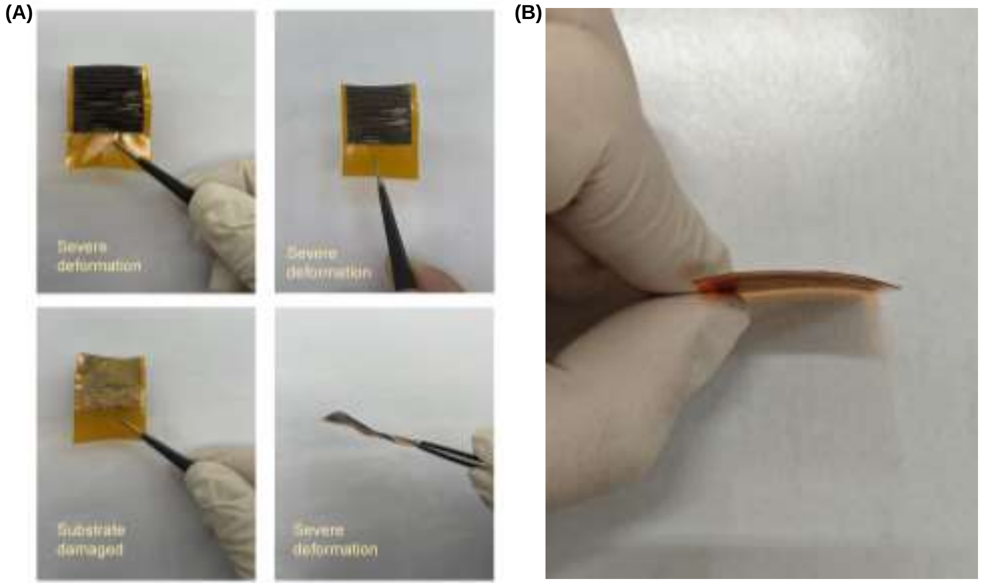

Figure S8. Photographs of the EMI shielding switch prepared under the laser power of 8 W (A) and 7 W (B).

Note S5: We conducted extensive parameter studies to identify conditions – specifically 3–7 W laser power–for LIG synthesis without film deformation. A laser power of 8 W is of limited practical value, as the excessive energy results in substantial deformation in prepared film (**Figure S8**) and causing significant measurement errors in both thickness and conductivity. A laser power of 3 W provides insufficient energy to form LIG, resulting in almost no change on PI surface.

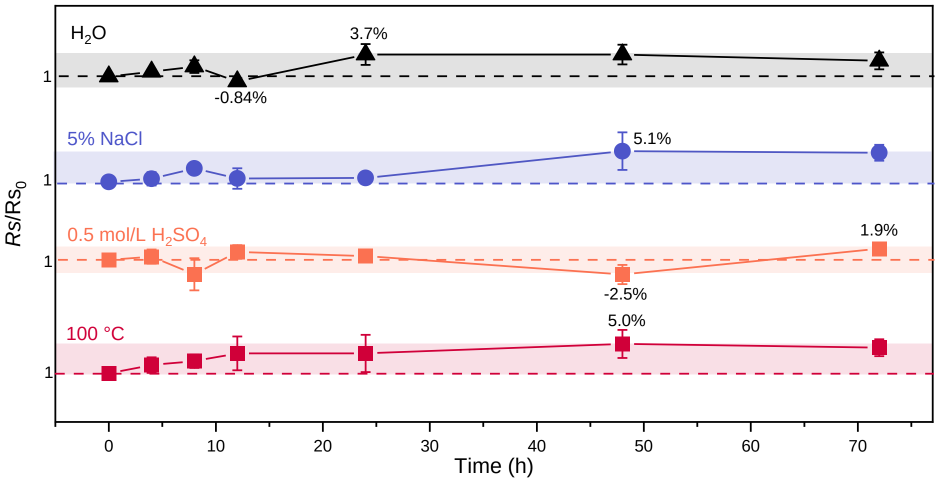

Figure S9. The change rate of electrical conductivity under conditions of H_2O, NaCl, H_2SO_4, and 100 °C.

To assess the stability of the prepared LIG, samples were exposed for 72 h to four representative environments: H_2O, 5 wt% NaCl solution, 0.5 M H_2SO_4, and air at 100 °C. As shown in **Figure S9**, electrical conductivity varied by less than 5.2%, demonstrating the potential of LIG for applications in extreme environments.

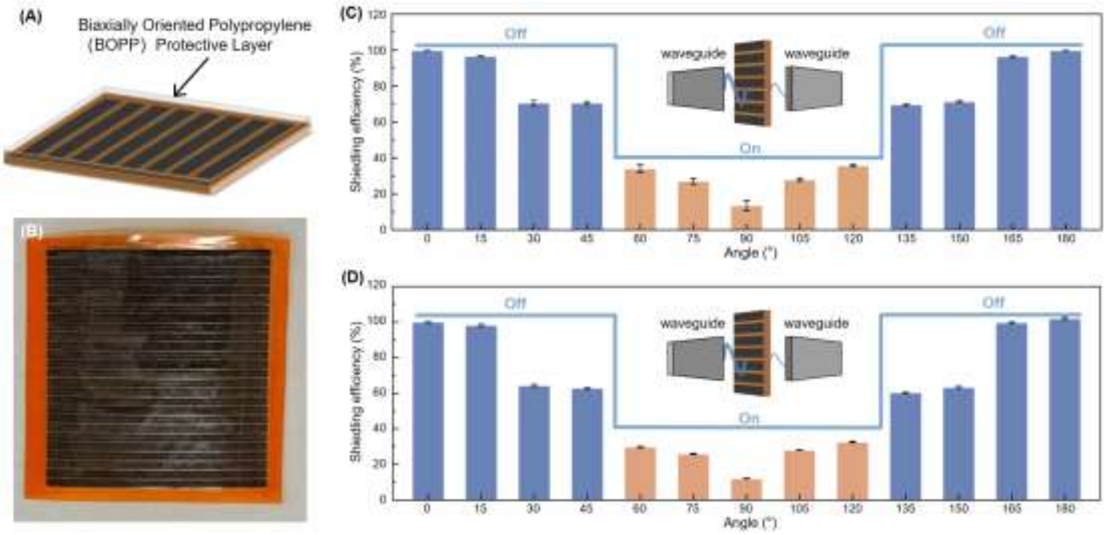

Figure S10. (A) Scheme and (B) photograph of the LIG metasurface protected by BOPP film. EMI shielding efficiency of (C) LIG metasurface and (D) LIG metasurface protected by BOPP film in various rotating angles at X-band.

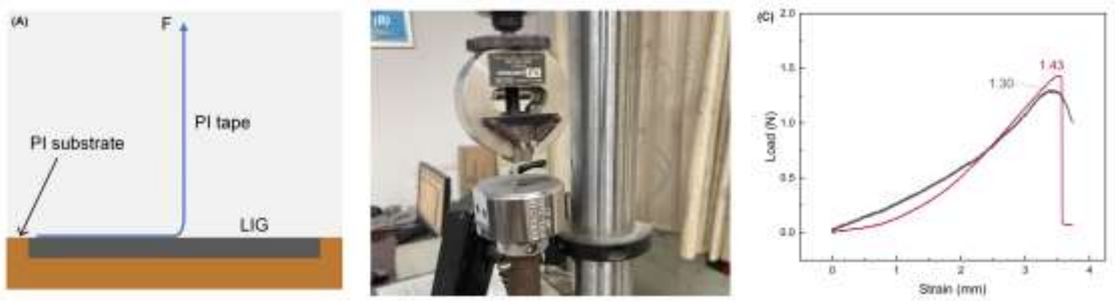

Figure S11. (A) Scheme, (B) photograph, and (C) results of the LIG peel strength test.

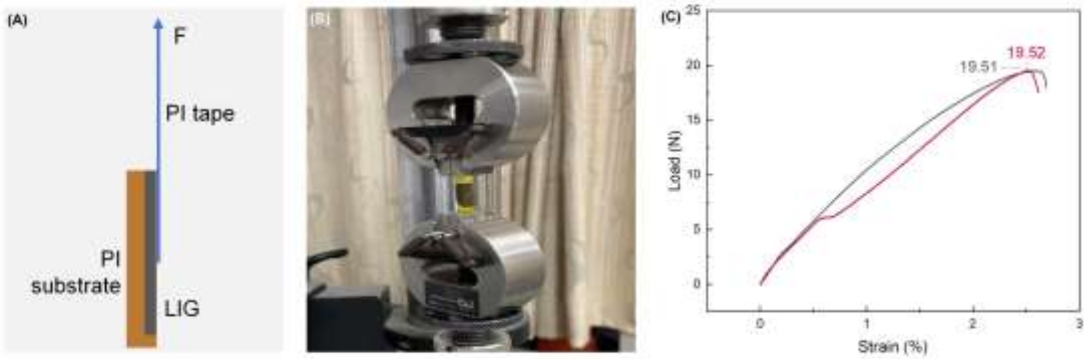

Figure S12. (A) Scheme, (B) photograph, and (C) results of the LIG shear strength test.

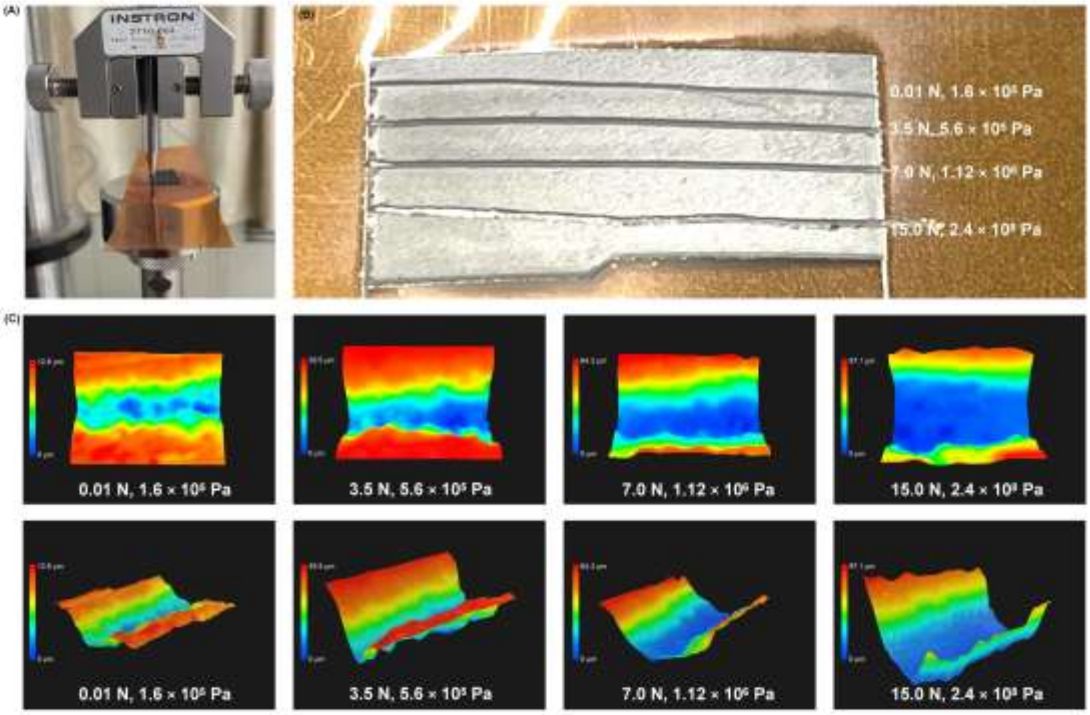

Figure S13. (A) Scheme, (B) photographs, and (C) super-depth-of-field images of scratching test.

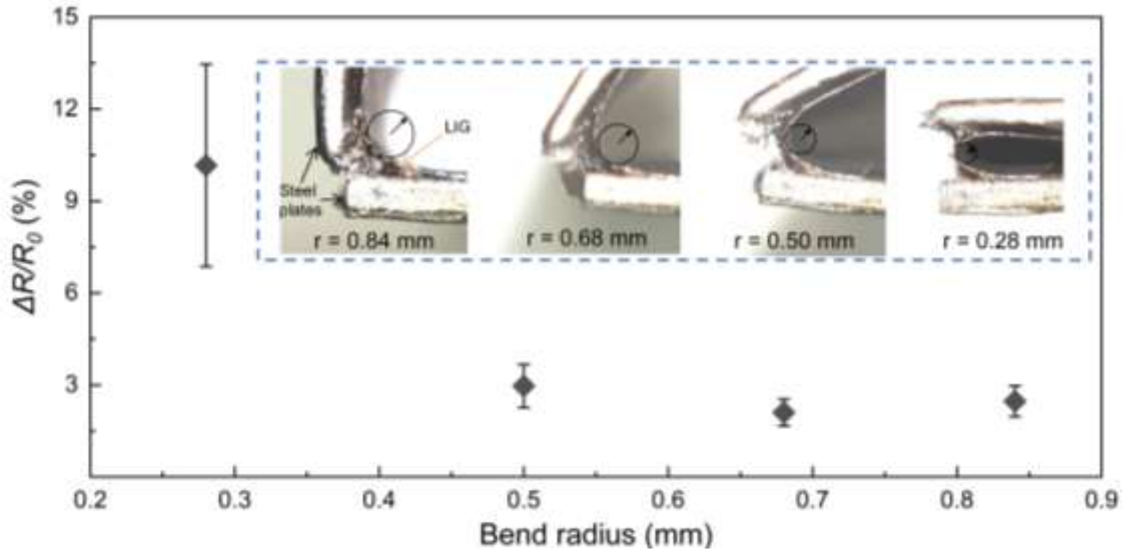

Figure S14. Resistance change rate of LIG in various bend radius.

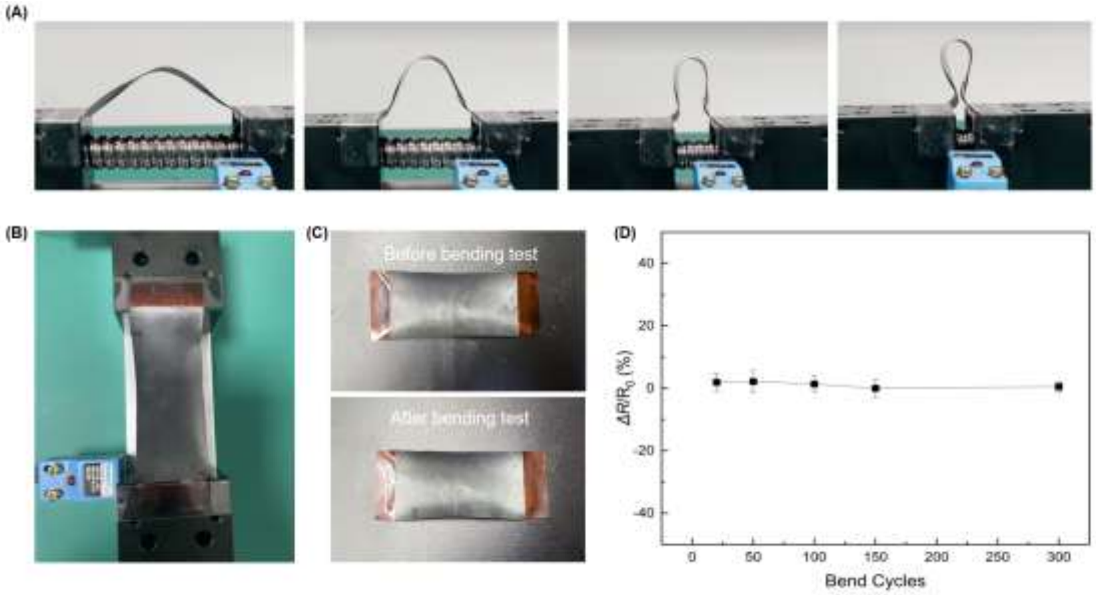

Figure S15. (A) Photographs of LIG with various bending degree. (B) Top view photographs of bending test. (C) photographs of LIG before and after bending test. (D) Resistance change rate of LIG during the bending cycle test.

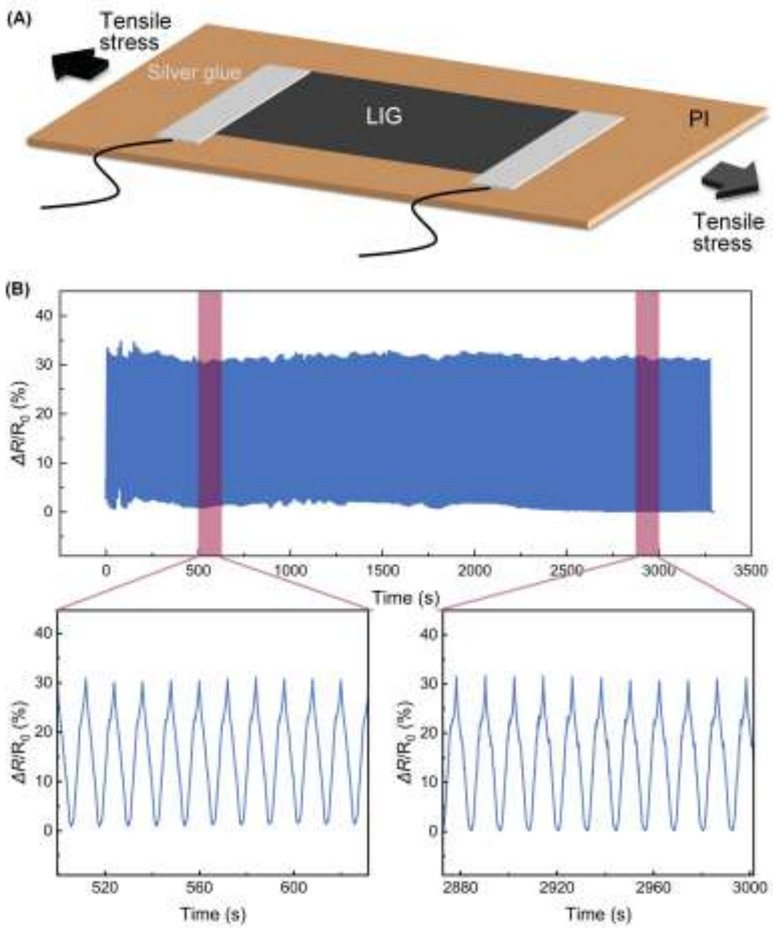

Figure S16. **(A) Scheme and (B) resistance change rate of the LIG tensile cycle test.**

Note S6: In order to better meet the practical application requirements, we used biaxially oriented polypropylene (BOPP) as a protective layer to encapsulate the LIG surface, as shown in **Figure S10A** and **S10B**. First, the EMI shielding efficiency of the LIG metasurface (**Figure S10C**) and the LIG metasurface protected by BOPP film (**Figure S10D**) at various rotational angles in X-band remains almost unchanged, indicating that the BOPP film packaging strategy does not affect the functionality of LIG metasurface. Second, the peel strength of the LIG metasurface with a width of 10 mm, was tested at an angle of 90 ° and a peeling speed of 0.5 mm/min (**Figure S11A** and **S11B**). As shown in **Figure S11C**, the average maximum peeling force of the LIG metasurface is 1.37 N, and the peel strength is 137 N/m. Thirdly, the shear strength of the LIG metasurface with the area of 60 mm^2 has been tested with the tensile speed of 0.5 mm/min (**Figure S12A** and **S12B**). As shown in **Figure S12C**, the maximum shear force of the LIG metasurface is 19.5 N and the shear strength is 325.2 kPa. Fourth, the scratching test have been conducted at the pressure of 1.6×10^5 Pa, 5.6×10^5 Pa, 1.12×10^6 Pa, and 2.4×10^8 Pa (**Figure S13A** and **13B**). **Figure S13C** shows that scratch depth and width increase with pressure until the LIG is completely removed. Fifth, bending radius was determined by mounting the sample between two steel plates and incrementally decreasing the inter-plate angle (**Figure S14**). The $\Delta R/R_0$ was measured after 50 bending cycles. Results show a minimum bend radius of 0.5 mm with $\Delta R/R_0 \approx 3.0\%$. Since conductivity directly affects electromagnetic shielding, a bend radius of 0.28 mm, corresponding to $\Delta R/R_0 \approx 10.2\%$, fails to meet application requirements. Sixth, the bending cycle test of the LIG metasurface with

300 cycles was carried out (**Figure S15A**). As shown in **Figure S15B**, the $\Delta R/R_0$ of the LIG metasurface remain almost 0, showing the high bending cycle stability of the LIG metasurface. Seventh, the tensile strain cycle test (with a stretching range of 0.5 mm, tensile strain of 0.72% and tensile speed of 5 mm/min) on the LIG metasurface for 560 cycles was conducted (**Figure S16A**). As shown in **Figure S16B**, the $\Delta R/R_0$ of the LIG metasurface remain almost unchanged, exhibiting the high tensile cycle stability of LIG metasurface.

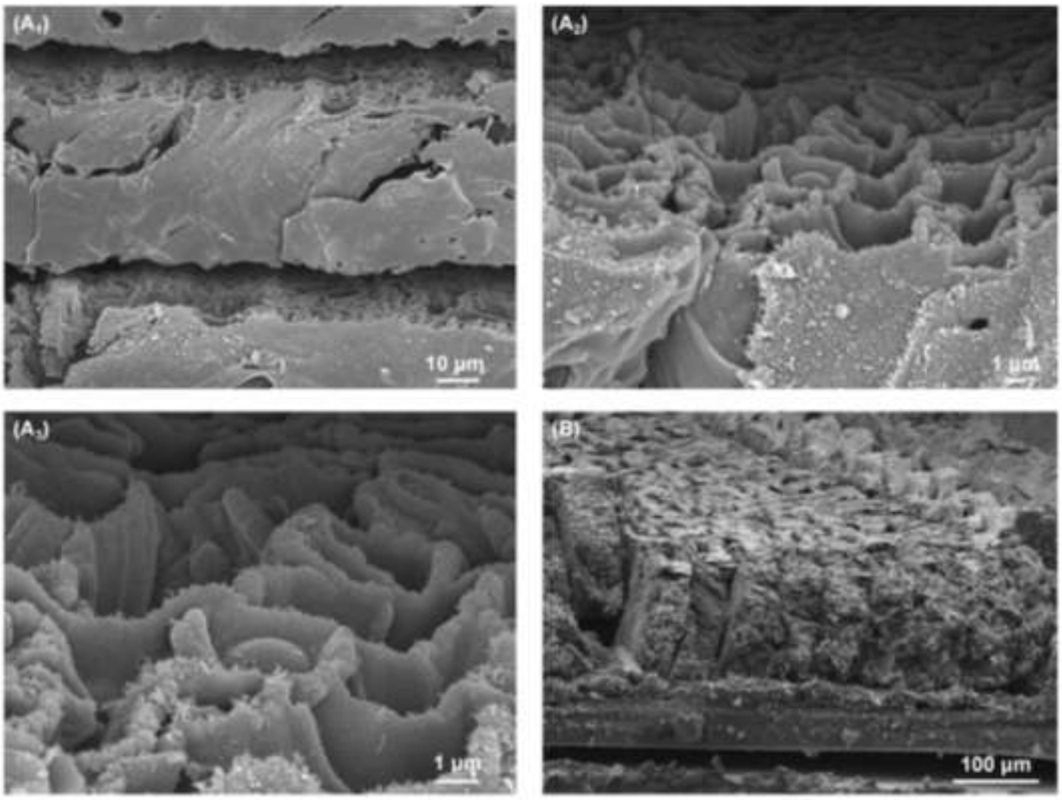

Figure S17. SEM images of surface (A) and cross section (B) of LIG prepared by laser-focused scanning.

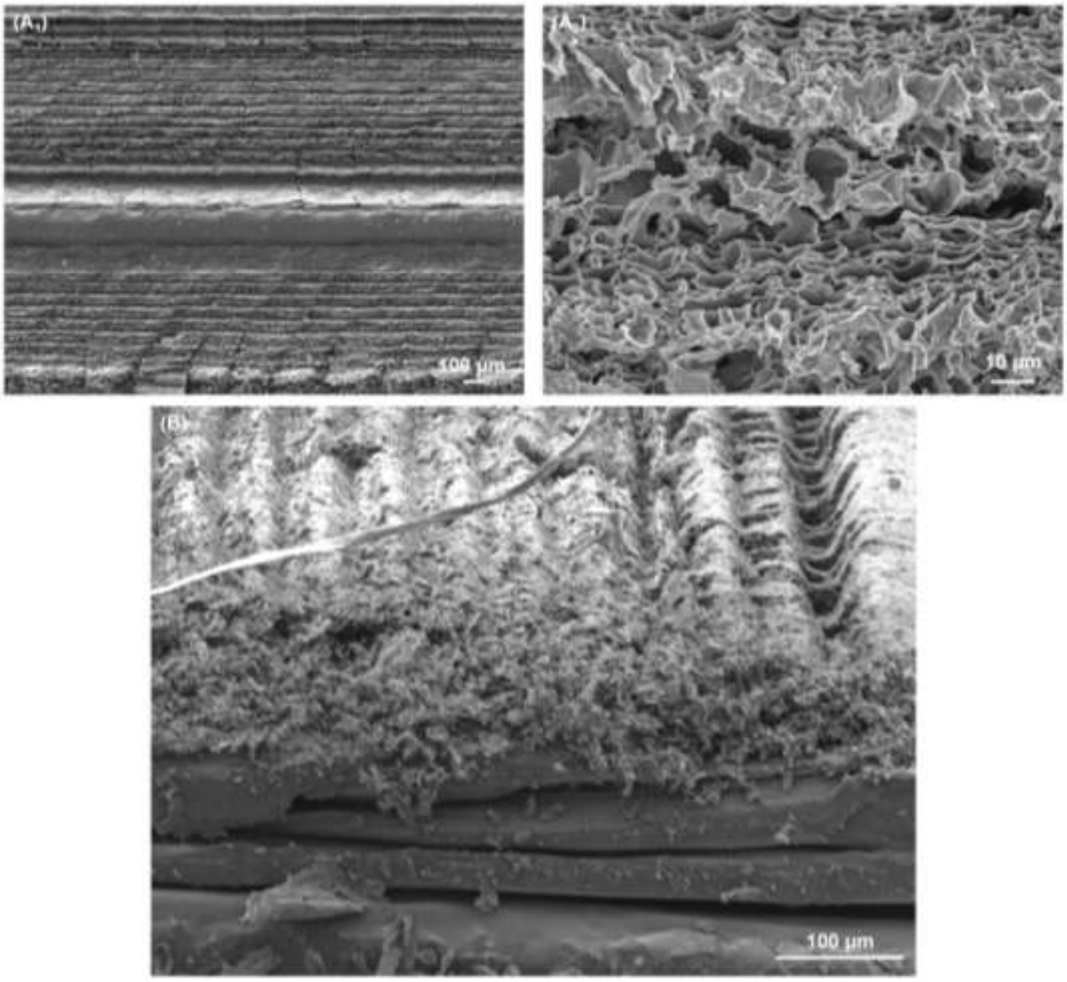

Figure S18. SEM images of surface and cross section LIG prepared by laser-defocused scanning.

Note S7: The LIG and PI film substrate exhibit good bonding strength with nearly no LIG detached from PI. However, the brittleness of LIG make the cross-sectional sample preparation difficult for SEM characterization, leading to unclear cross-sectional morphology (**Figure S17B and S18B**). Even so, the groove structure with large breadth depth ratio in **Figure S17B** obviously demonstrating the effect of photoetching in laser-focused scanning is stronger than that in laser-defocused scanning.

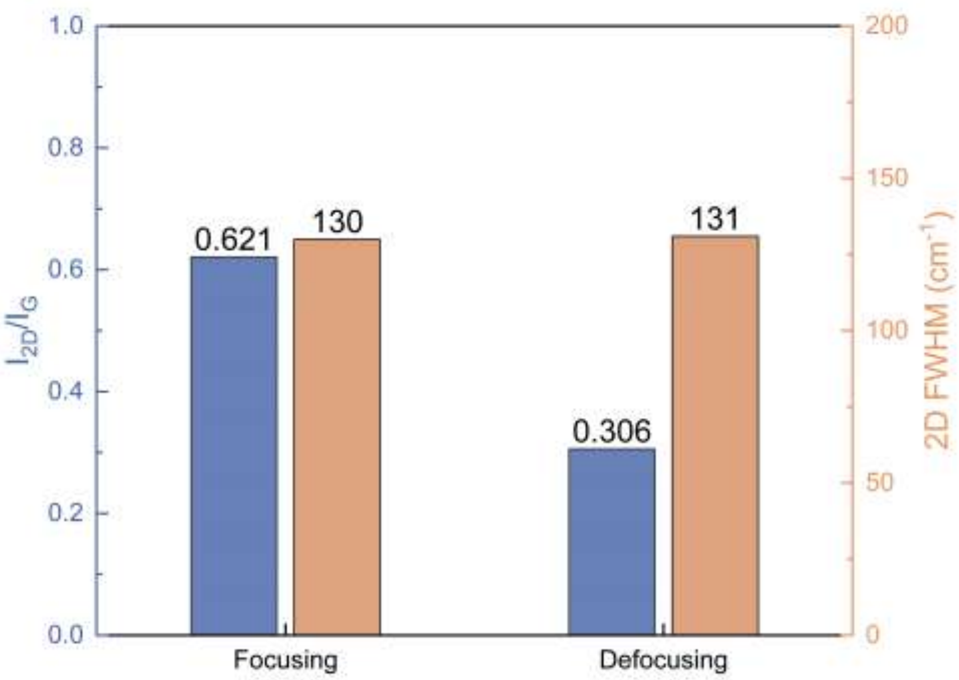

Figure S19. ***I_{2D}/I_G*** **and corresponding full width at half maximum (FWHM) of the 2D band calculated from the Raman spectroscopy.**

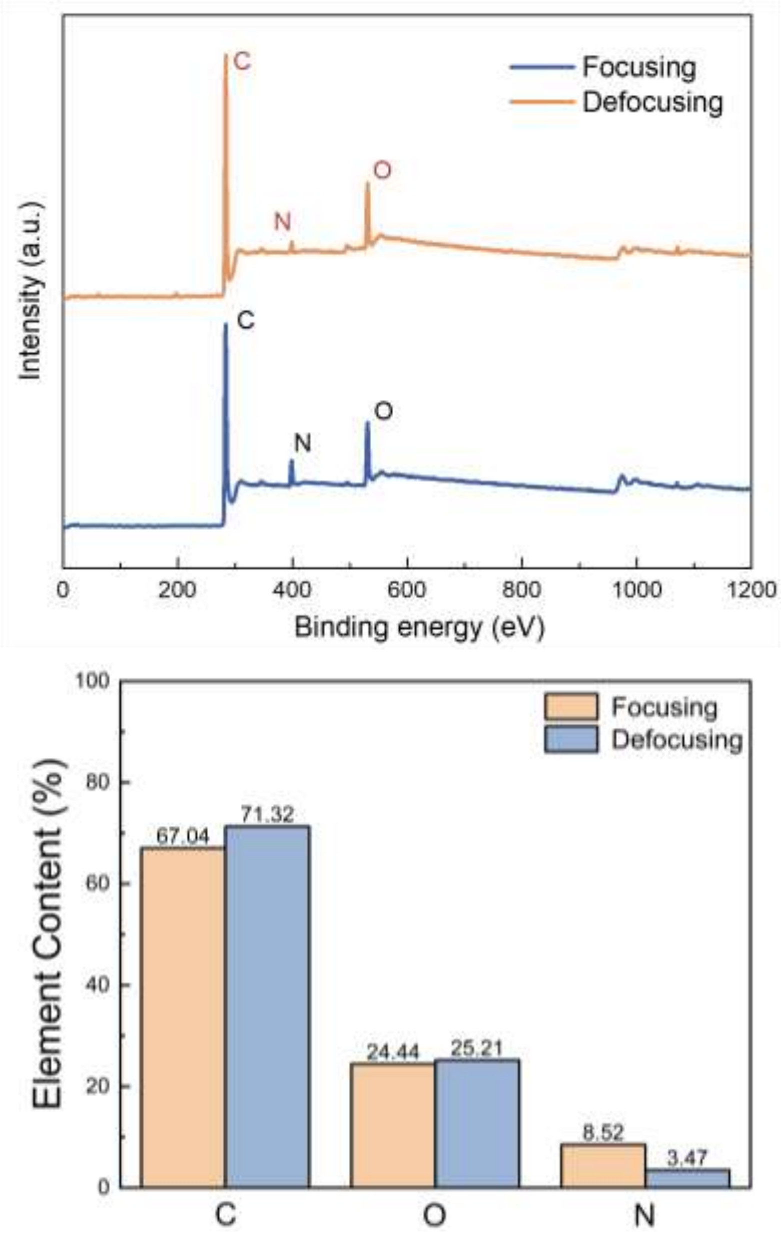

Figure S20. X-ray photoelectron spectroscopy (XPS) and calculated element content.

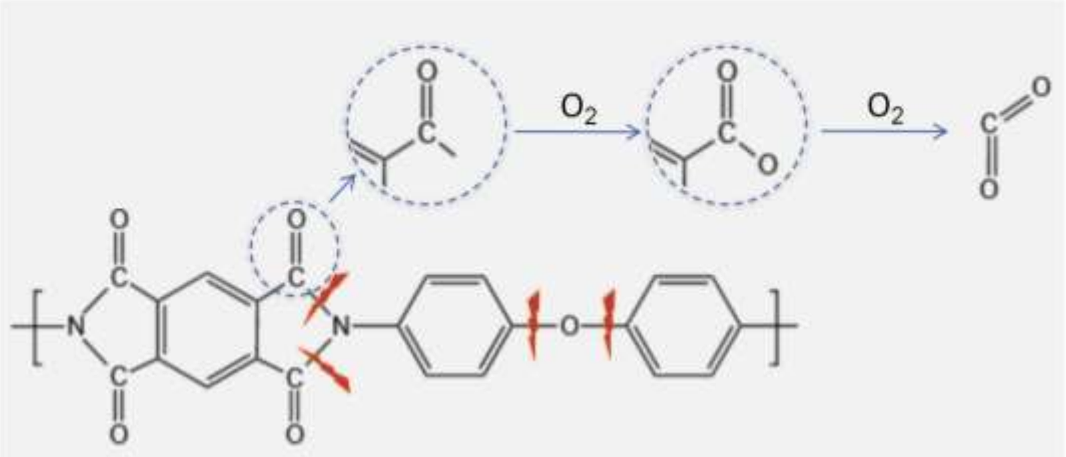

Chemical bond	Bond length (10^{-12}m)	Bond energy (kJ/mol)
C=O	120	728
C—C	154	332
C—N	148	305
C—O	143	326
—O—	140	240

Figure S21. Schematic diagram of the laser-induced reaction.

Note S8: **Figure S21** gives a schematic illustrating the formation of CO_2 from N-C=O. The key bond energy of C-N is relatively low thus preferentially dissociated during the reaction, leading to the hanging key reacted with O_2 in the air[11, 55]. Consequently, no C-N bonds were detected in the XPS results of F-LIG and DF-LIG. The increased C-O bonds in F-LIG (**Figure 2G**) may result from unreacted bonds in PI and the enhanced photoetching effect, which induces C dangling bonds that react with oxygen molecules in the air. The last step of O-C=O to O=C=O need more induced energy, thus remaining unreacted O-C=O in laser-focused scanning.

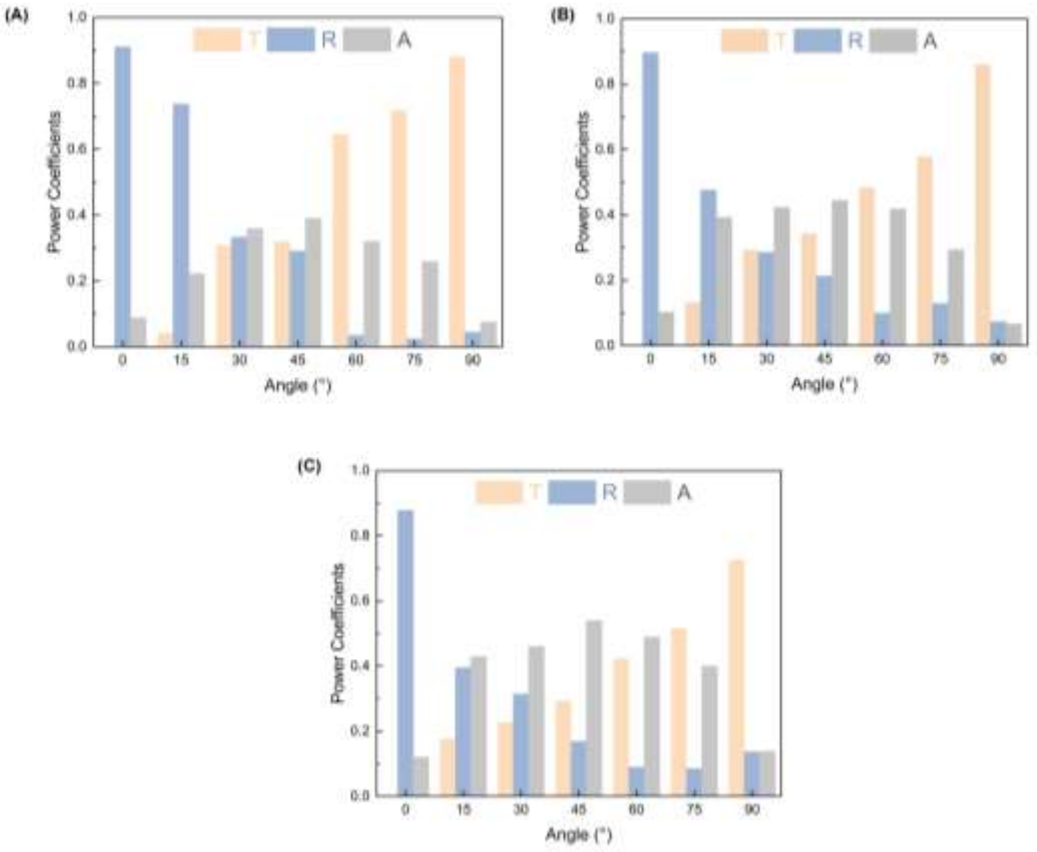

Figure S22. Coefficients of transmission (*T*), reflection (*R*), and absorption (*A*) of the EMI shielding switch at X-band (A), Ku-band (B) and K-band (C).

Note S9: In electromagnetic shielding characterization, $T = P_t/P_i$, $R = P_r/P_i$, and $A = P_a/P_i$, where P_t represents the transmitted power, P_i is the incident power, and P_a is the absorbed power. T denotes the proportion of electromagnetic waves transmitted through the shielding material, describing its transmittance capability. R and A denote the proportion of electromagnetic waves reflected and absorbed by the shielding material, quantifying its reflection and absorption performance. For traditional EMI shielding materials, smaller value of T means better shielding performance and is expected. **Figure S22** illustrates the variation of coefficients with respect to the angles of the EMI shielding switch. As the angle increases, the value of T gradually rises, indicating a decreased ability to shield incident electromagnetic waves or an enhanced

ability to transmit them. The minimum value of *T* is at 0 ° (Off-state) and the maximum value is at 90 °(On-state), which is consistent with the discussion in **Figure 3A** and **3B**. The value of *R* basically exhibits a downward trend with the angle increasing. We suppose it is contributed to two reasons: (1) The electrical conductivity perpendicular to the laser canning direction is lower than that parallel to the scanning direction in LIG. Even if the metasurface switch is considered to be a complete shielding film material, the value of *R* will exhibit a similar trend. (2) The incident electromagnetic waves induced electron oscillations decreased, thus secondary electromagnetic wave is reduced (discussed in detail in **Figure 3G** and **3H**). Interestingly, the value *A* of shows a trend of first rising and then falling with the angle increasing. It is well known that when a wave incident on the interface between material and air, part is reflected, while the rest enters the material, where it is either absorbed or transmitted. From 0 °to 45 °, the leading mechanism is assumed to be the following: the decrease in conductivity leads to fewer reflected waves and more waves entering, which are subsequently adsorbed. From 0 ° to 45 °, the induced electron oscillations decrease significantly, resulting in a very small kinetic energy gain for the electrons, which continues to decrease, leading to a downward trend in the value of *A*.

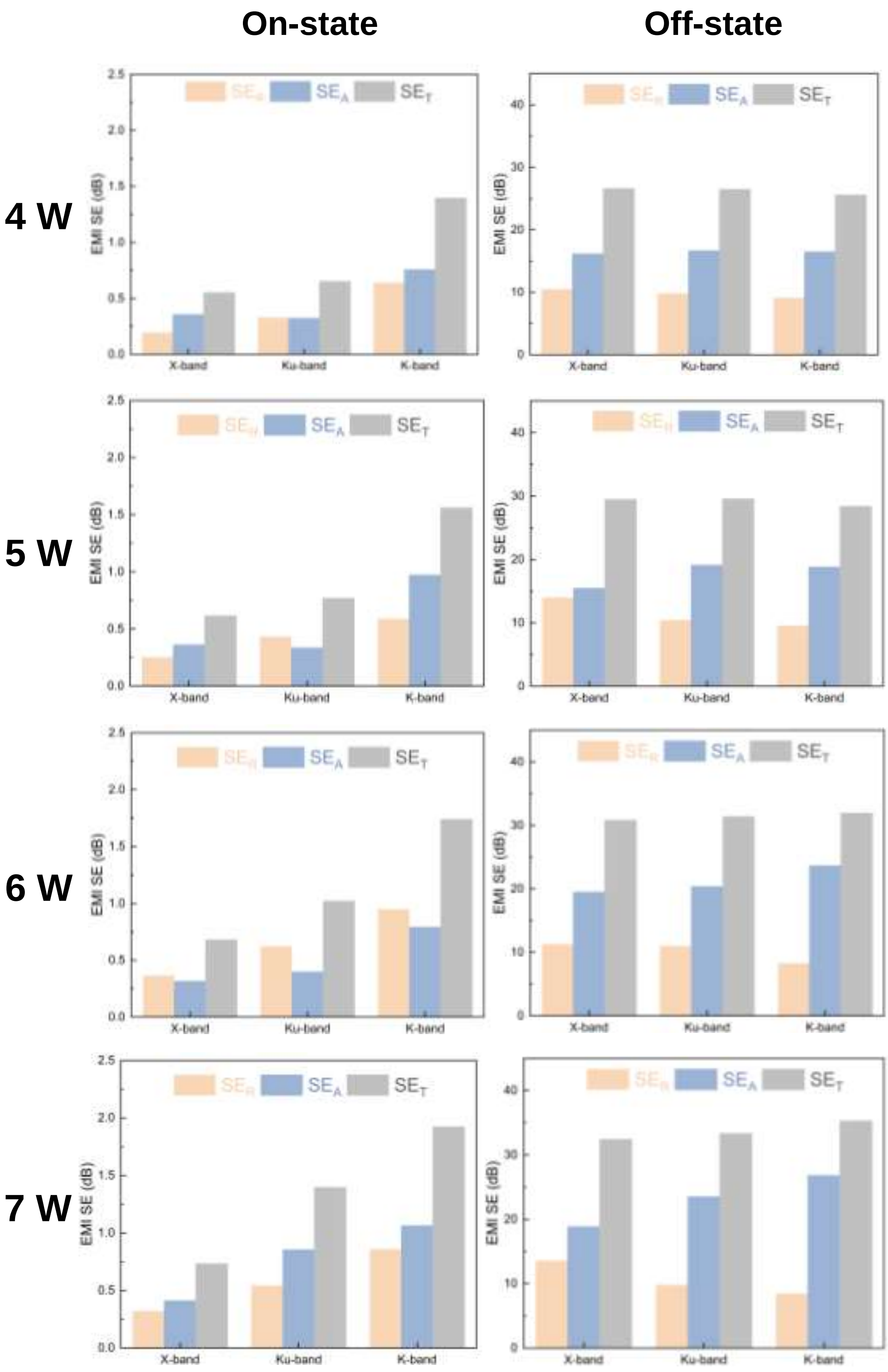
On-state
Off-state
4 W
5 W
6 W
7 W
EMI SE (dB)
SE_R
SE_A
SE_T
X-band
Ku-band
K-band

8 W

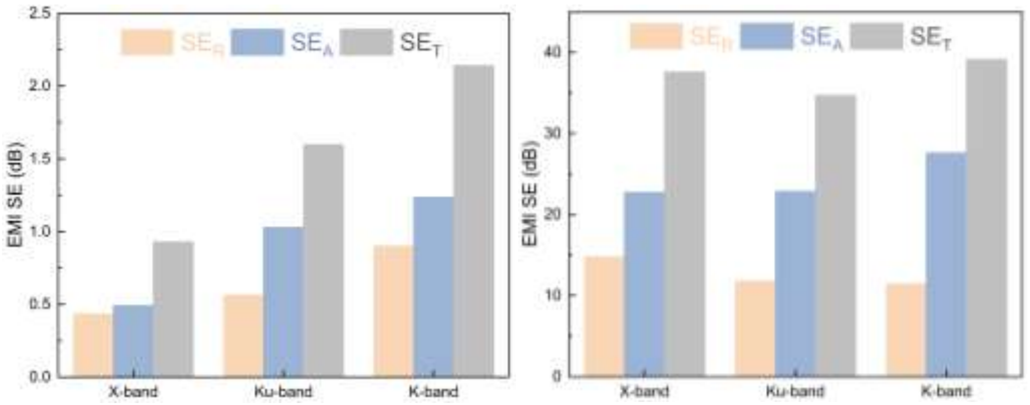

Figure S23. ***SE_T*, *SE_A* and *SE_R* at various electromagnetic wave band and laser processing power.**

Note S10: SE_A and SE_R reflect the material's capability to reflect and absorb electromagnetic waves. SE_T represents the total shielding effectiveness, comprising SE_A and SE_R. As exhibited in **Figure S23**, under On-state SE_T increases with incident waves changing from X-band to K-band, reflecting stronger electron oscillations at higher frequencies. By contrast, under Off-state, SE_T remains nearly constant across frequency bands. Conversely, SE_A and SE_R exhibit opposite trends as frequency increases. Under Off-state, the skin effect intensifies, increasing surface impedance and reducing impedance mismatch with air, thereby decreasing reflection (i.e. SE_R declines). Moreover, because the waveguide-port separation is fixed, higher frequencies (shorter wavelengths) incur greater attenuation through the same thickness, increasing SE_A.

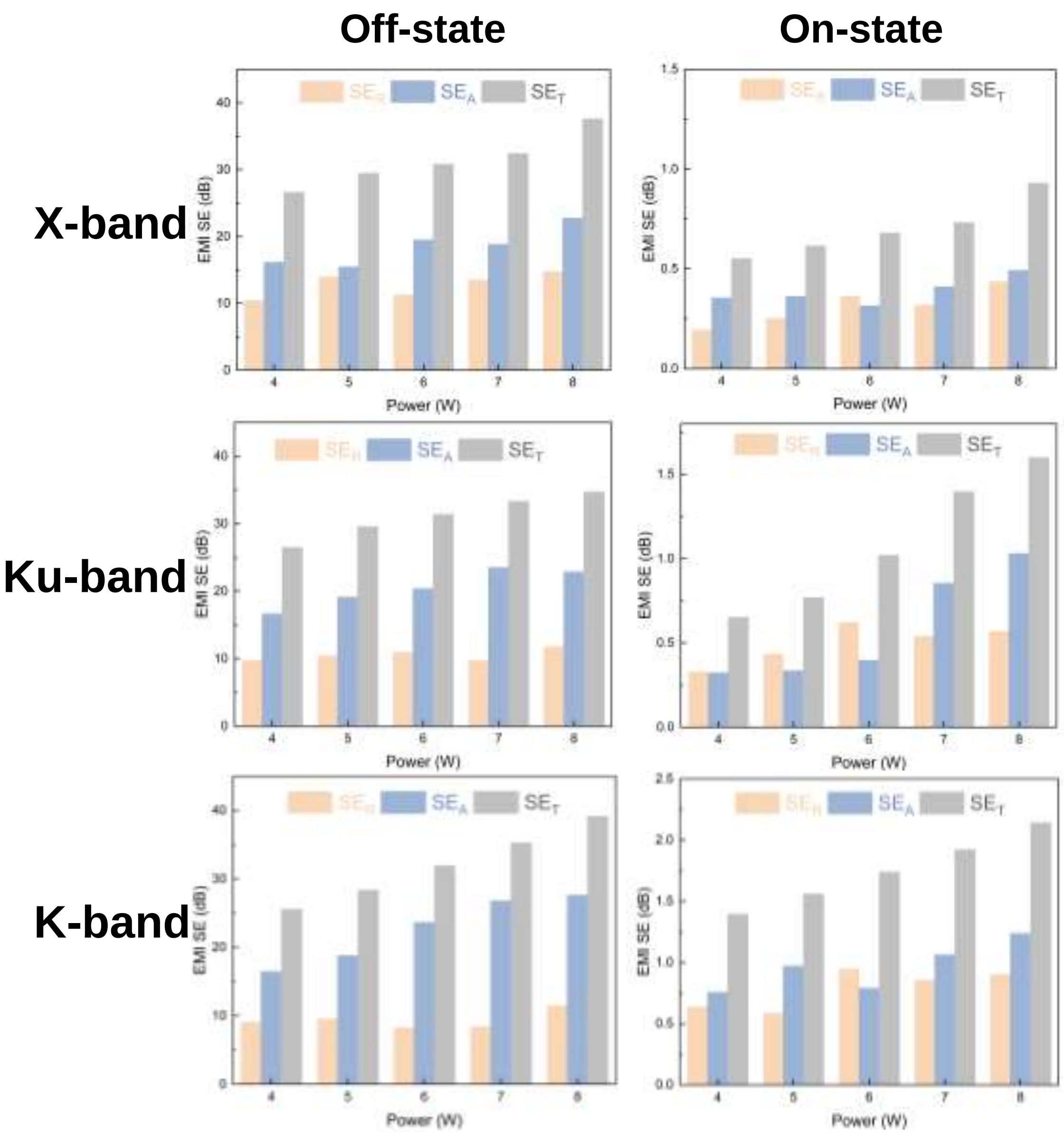

Figure S24. ***SE_T*, *SE_A* and *SE_R* of EMI shielding switches at various electromagnetic wave band and laser processing power.**

Note S11: Upon the On-state of the EMI shielding switch, SE_R reaches its highest value at a laser power of 6 W (8 W) under the incident wave frequency of Ku-band and K-band (X-band) (**Figure S24**). The wave-induced electron oscillations are relatively weak (On-state), thus the influence of electrical conductivity on the number of free electrons is obvious. **Figure 2C** exhibits the conductivity of LIG fabricated by

6 W laser scanning is the highest, which aligns with the previously explained mechanism for Ku-band and K-band waves. The highest value of SE_R occurs at a laser power of 8 W under X-band and may be attributed to the measurement error induced by significant deformation and substrate damage. Across laser powers in the Off-state, SE_R values converge, indicating that minor conductivity variations exert a limited influence. Meanwhile, with increasing laser power, SE_T exhibits an overall upward trend, likely owing to the increased thickness of LIG, which leads to enhanced electromagnetic wave attenuation.

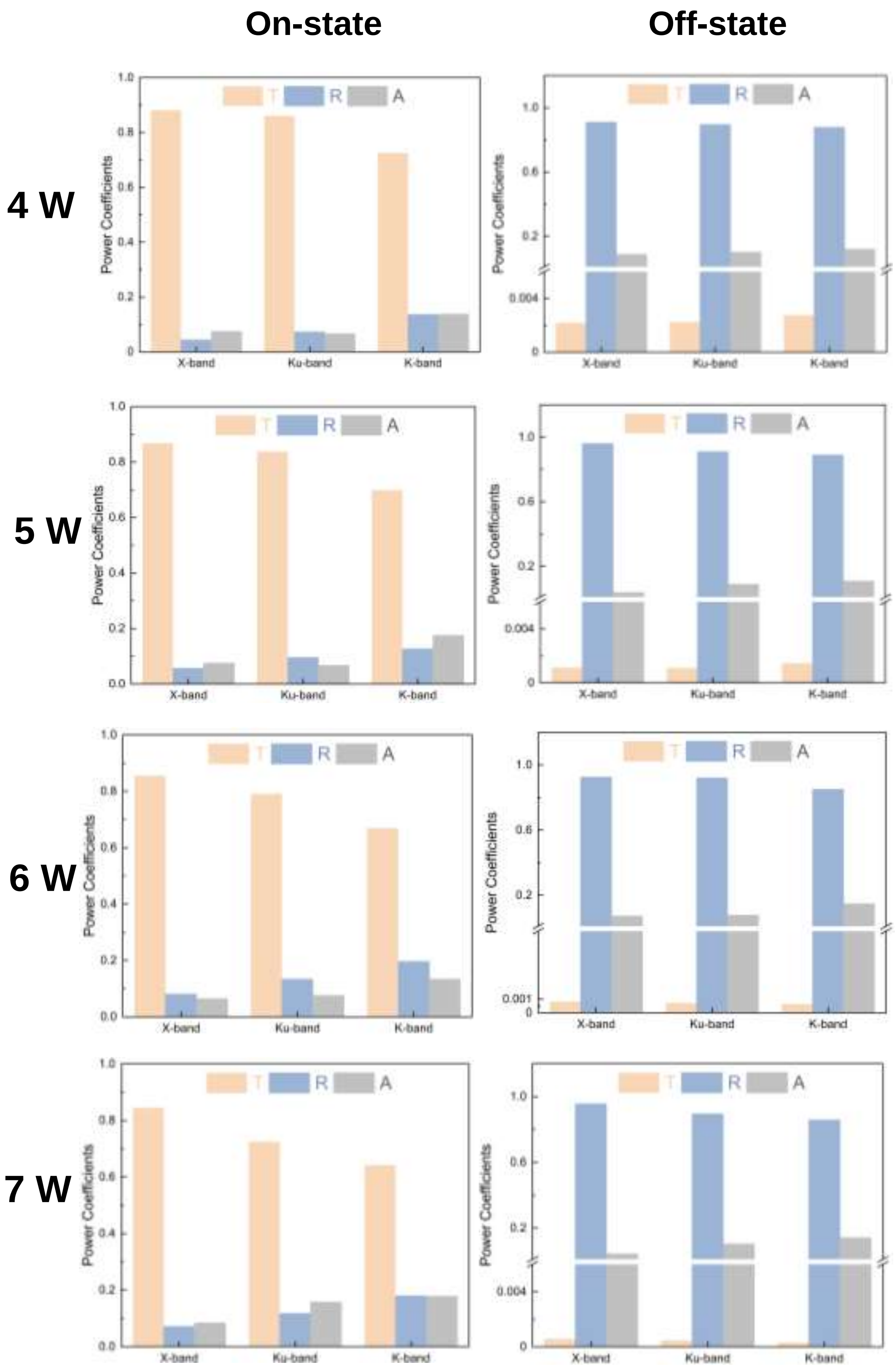
On-state
Off-state
4 W
5 W
6 W
7 W
T
R
A
Power Coefficients
X-band
Ku-band
K-band

8 W

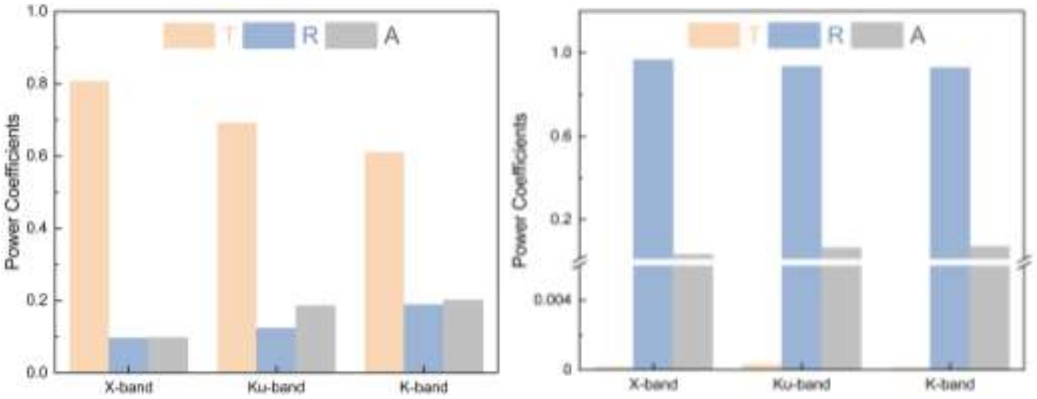

Figure S25. Coefficients of *T*, *R*, and *A* of EMI shielding switches at various electromagnetic wave band and laser processing power.

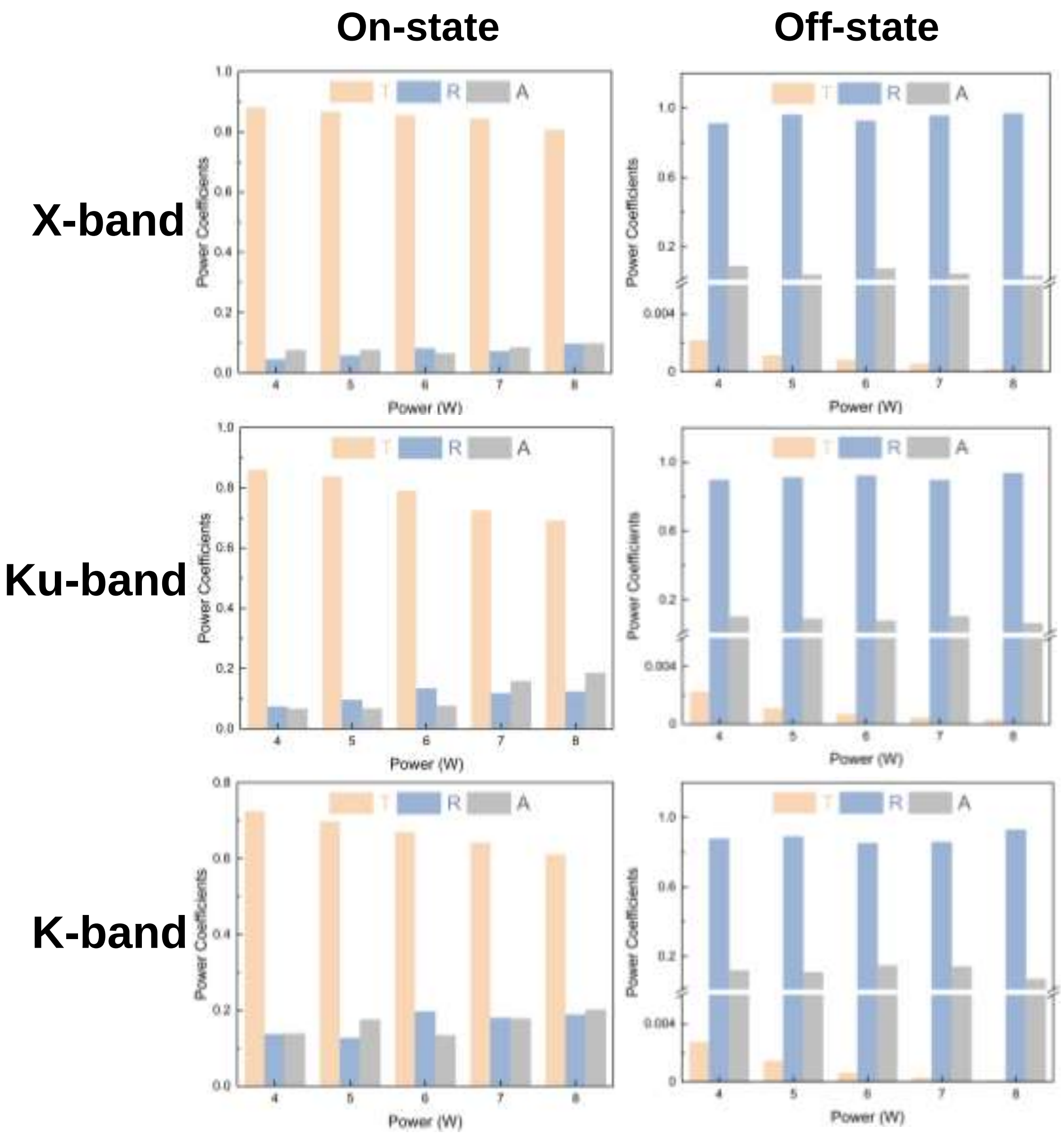

Figure S26. Coefficients of reflection, absorption and transmission of EMI shielding switches at various electromagnetic wave band and laser processing power.

Note S12: When considering whether the EMI shielding is absorption- or reflection-dominant, coefficients of *T*, *R*, and *A* are recommended as measure, instead of SE_T, SE_R, and SE_A Therefore, the switch fabricated in this work is a reflection dominant EMI shielding material.

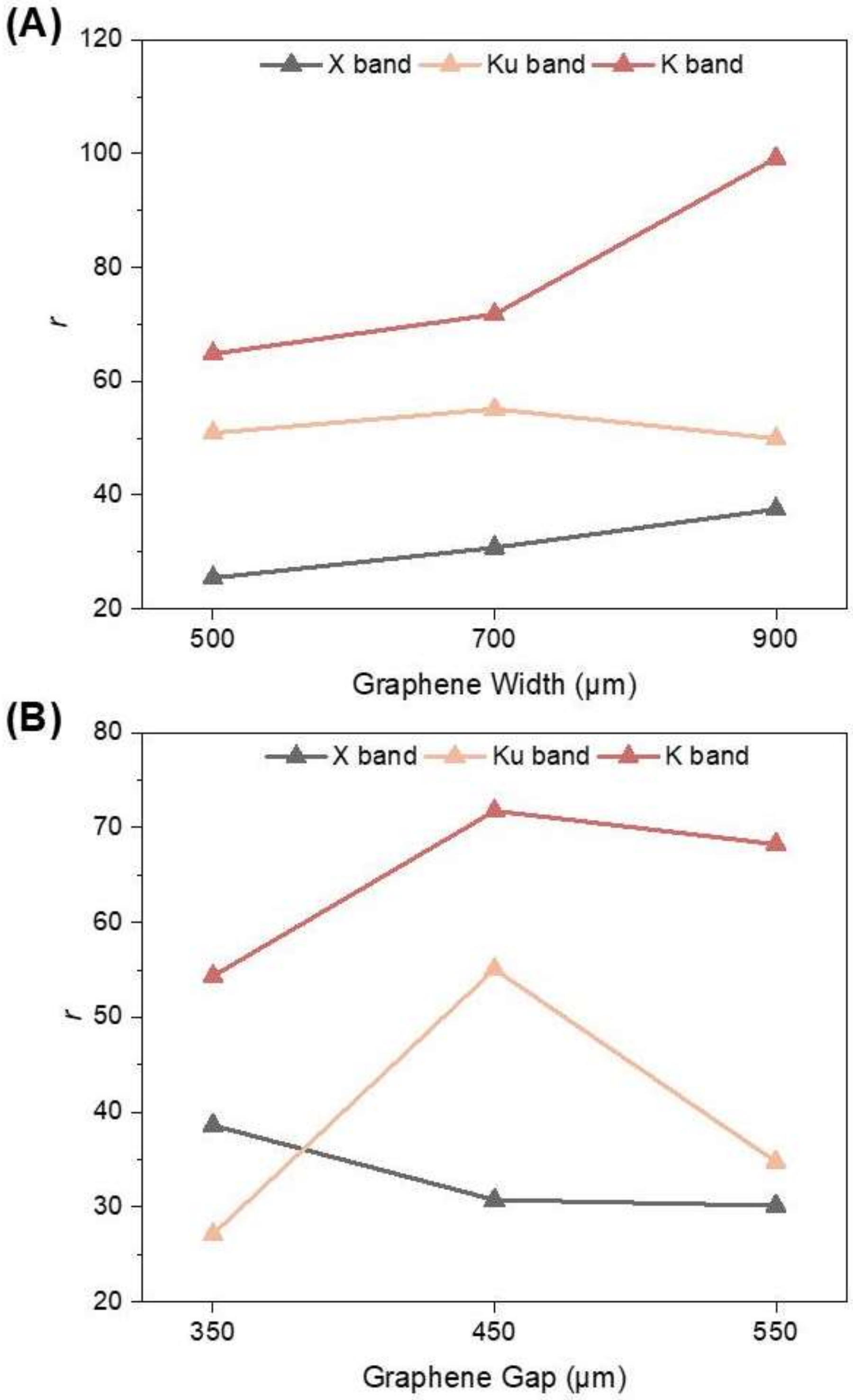

Figure S27. ***r*** **values of EMI shielding switches with various LIG width and gap width.**

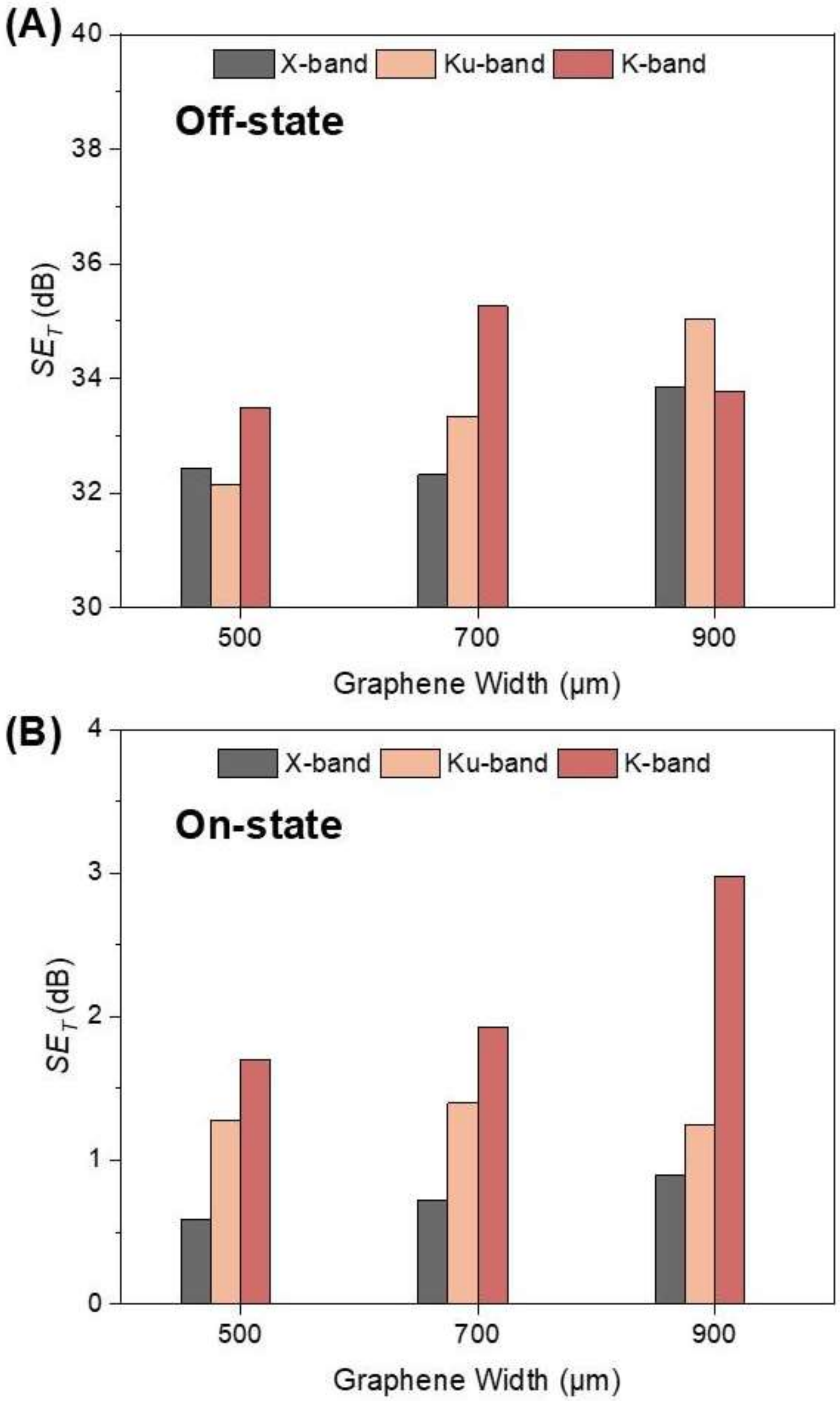

Figure S28. ***SE_T*** **of EMI shielding switches with different LIG width at off-state (A) and on-state (B).**

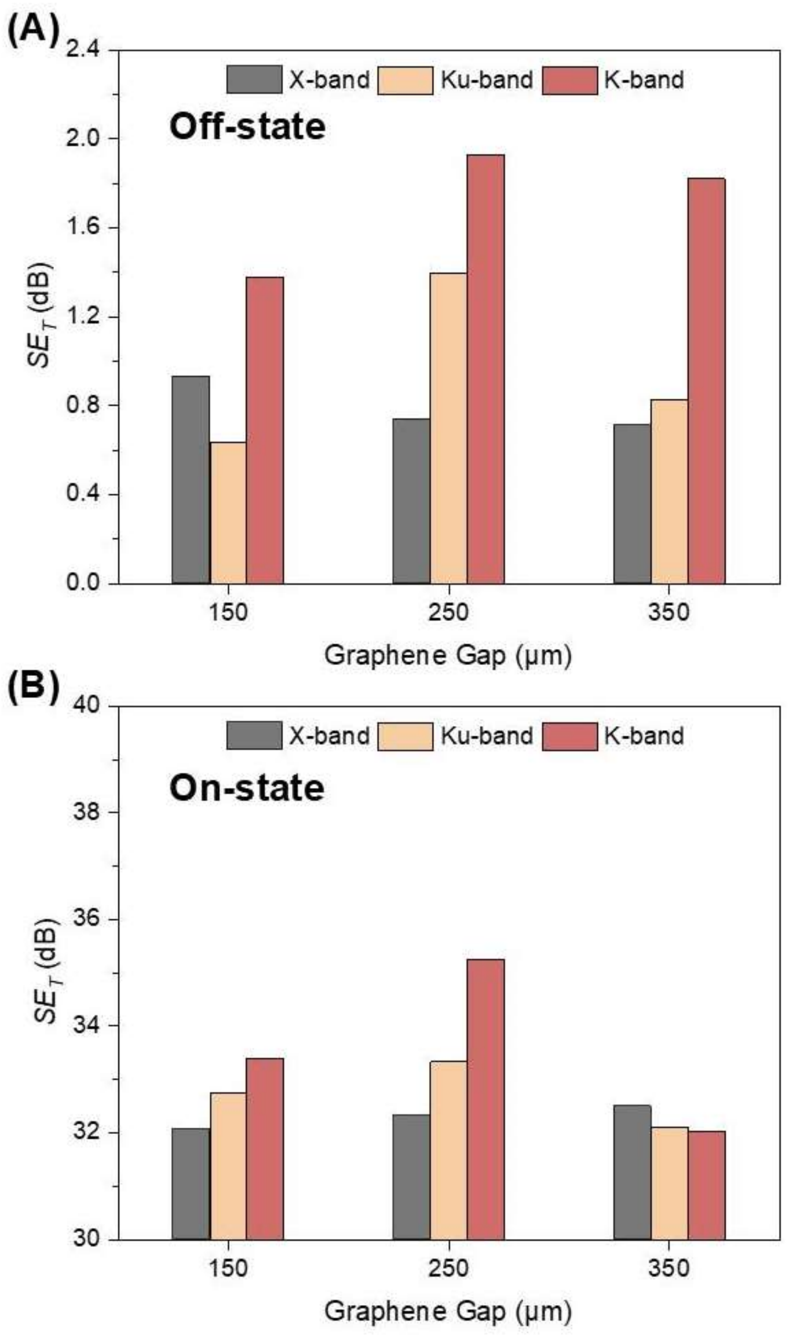

Figure S29. ***SE_T*** **of EMI shielding switches with various gap width at off-state (A) and on-state (B).**

Note S13: Figure S27–S28 summarize the effect of LIG width and gap width on EMI SE_T and r (reconfigurability factor). Under Off-state of the EMI shielding switch, the LIG width has minimal impact on SE_T, and the value consistently exceeding 30 dB. For instance, under X-band wave, the difference between the maximum SE_T (33.855

dB) and the minimum SE_T (32.320 dB) is only 1.535 dB, representing a 4.534% change. Under On-state of the EMI shielding switch, the LIG width shows great impact on SE_T. For instance, under X-band wave, the difference between the maximum SE_T value (18.745% / 0.902 dB) and the minimum (12.710% / 0.590 dB) is 6.035%, or 0.312 dB, corresponding to a 32.195% / 34.590% change. In the Off-state of the EMI shielding switch, the gap width has minimal impact on SE_T, with the value consistently exceeding 30 dB. For instance, under X-band wave, the difference between the maximum SE_T value (32.490 dB) and the minimum (32.062 dB) is only 0.428 dB, representing a 1.317% change. In the On-state of the EMI shielding switch, the gap width shows great impact on SE_T. For instance, under X-band wave, the difference between the maximum SE_T value (19.300% / 0.931 dB) and the minimum (15.064% / 0.709 dB) is 4.236%, or 0.222 dB, corresponding to a 21.948% / 23.845% change. Since r evaluates the comprehensive On/Off performance, the SE_T value in the On-state plays a more crucial role in determining r. The calculation formula for r is discussed in **Figure 3F**, and the r values from previous studies and this work are presented in **Table S3**.

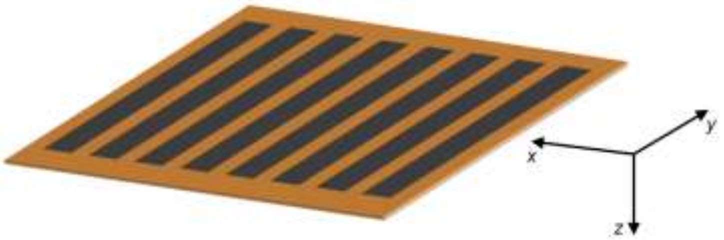

Figure S30. Scheme of the x, y, and z directions in the CST simulation.

Note S14: In the CST simulation, the line width, inter-line gap width, and conductivity of LIG were set to 700 μm, 350 μm, and 340.2 S m^{-1}, respectively. The thickness of LIG and PI substrate were set to 45 μm and 150 μm, respectively.

Table S3. Statistical table of electromagnetic shielding switches' synthesis time and *r* value in others work.

No.	Type of tunability	Materials	Thickness (mm)	Synthesis duration (h)	r^1	Ref.
1	Compression	Wood-derived carbon/XC-72 nanoparticles aerogel	> 70	> 52	27.56	[21]
2		TPI-MXene/carbon foam	10	> 124	99.68	[25]
3		EVA@PPy@Ag foam	2	> 13	76.18	[26]
4		Melamine foam@MXene/Ag nanowire sponges/PEG	12	> 60	94.25	[27]
5		WF/CNTs composite foam	16	> 86	82.22	[22]
6		PU/CNTs/TPI	10	> 6	62.85	[23]
7		Melamine foam@Ag/PDA/CNT waterborne polyurethane	4	> 65	99.68	[28]
8		Patterned anisotropic magnetic liquid metal/PDMS	/	>4	80.05	[29]
9		PU/RGO foam-5%	15	> 4	96.61	[24]
10		PU/RGO foam-10%	15	> 4	99.54	[24]
11	Stretching	pre-linked Ni chains elastomer	0.3	> 13	49.88	[32]
12		CNT@liquid metal/polyacrylamide/gelatin	4	> 3	/	[56]
13		Fe/liquid metal/PDMS	0.2~0.78	> 8	99.13	[33]
14		3D liquid metal network	1-2	/	99.99	[34]
15		Liquid metal elastomer composite (BiInSn)	2~3.6	> 10	16.82	[35]
16		Liquid metal elastomer composite (Ga)	0.1~0.5	> 10	92.06	[35]
17		Ag nanoparticles/SEBS	3.8	1.25	99.84	[36]
18		CNT/TPU	2	> 1	94.75	[30]

19		Liquid metal GaIn24.5/Ni	0.05	> 1	90.00	[37]
20		PEDOT:PSS/ waterborne PU	0.15~2	> 24	99.99	[31]
21	Temperature	VO_2/CNF	0.6~1.9	24	98.93	[38]
22		core–shell structural PNIPAM@p-PDA biomicrospheres	0.02~0.07	24	99.84	[39]
23	Humidity	VO_2/EPM composites foam	3.5	0.5	98.66	[40]
24		VO_2/poly(vinylidene fluoride-cohexafluoropropylene)	0.15~1.80	50	35.44	[41]
25		$Ti_3C_2T_x$-WVO_2	2	> 12	99.87	[43]
26		RGO/VO_2-300℃	2.82	30	87.70	[42]
27		RGO/VO_2	5.8	30	90.00	[42]
28	Electrochemical potential	Pyrolytic graphite-wet RGO/CNTs/PP non-woven spacer-pyrolytic graphite	2	10	36.90	[44]
29		MXene film ($Ti_3C_2T_x$ electrode in 1 M H_2SO_4)/PET	0.0006	> 44	94.31	[45]
30	Rotation	MXene film (V_2CT_x electrode in 1 M H_2SO_4)/PET	0.0006	> 44	99.70	[45]
31		MXene/CNF aerogels	2	/	94.99	[52]
32		MXene@wood	2	38	99.82	[53]
33		Polyvinyl butyral/Ni-graphene/short-cut CF composite films	0.2	9	36.90	[46]
34		CF reinforced polymer	0.55	1.5	90.00	[47]
35		CF hybrid fabrics	1	> 9	63.44	[48]
36		RGO@directional porous carbon	1	>15	98.01	[49]
37		CNT/NFC	2	/	99.96	[50]
38		Co based amorphous wires	0.117~0.127	55	12.90	[51]
39<br>40		LIG metasurface	0.0668	0.1	9.66[2]<br>11.77[3]<br>14.10[4]	This work

	27.53[5]

[1]: In the calculation formula of r, $SE_{max\%}$ and $SE_{max\%}$ correspond to two specific values, respectively. $SE_{max\%}$ refers to the maximum value of electromagnetic interference shielding efficiency in tunable shielding devices, which should be 100% under ideal conditions. Besides, $SE_{min\%}$ denotes the minimum of EMI SE, ideally fully wave-transparent, of 0.

[2]: r value at the frequency of 9.85 GHz with the best on/off EMI switch performance.

[3]: r value at the frequency of 8~12 GHz.

[4]: r value at the frequency of 12~18 GHz.

[5]: r value at the frequency of 18~26.5 GHz.

Trans-1,4-polyisoprene (TPI), ethylene-vinyl acetate copolymer (EVA), polypyrrole (PPy), poly(ethyleneglycol) (PEG), wheat flour (WF), carbon nanotube (CNT), polyurethane (PU), trans-1,4-polyisoprene (TPI), poly-dopamine (PDA), polydimethylsiloxane (PDMS), 3 dimension (3D), styrene-(ethylenebutylene)-styrene (SEBS), reduced graphene oxide (RGO)， thermoplastic polyurethane (TPU), poly (N-isopropylacrylamide)@porous polydopamine (PNIPAM@p-PDA), expanded polymer microsphere (EPM), poly (3,4-ethylenedioxythiophene) /poly (styrenesulfonate) (PEDOT:PSS), vanadium dioxide (VO_2), polyprolene (PP), poly (ethylene terephthalate) (PET), carbon fiber (CF), nano-brillated cellulose (NFC), cellulose nanofibrils (CNF), laser-induced graphene (LIG).

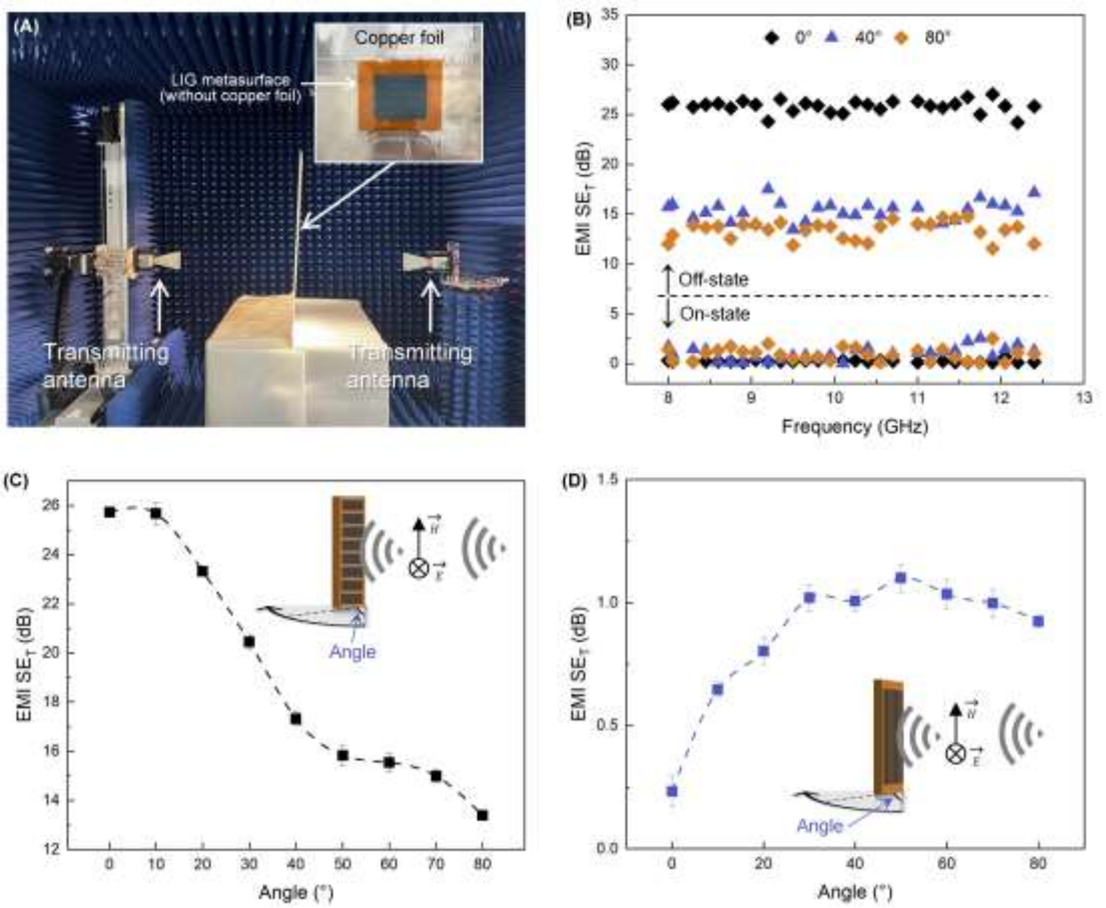

Figure S31. (A) Scheme of the oblique-incidence test. (B) SE_T at the incident angle of 0°, 40°, and 80°. Average value of SE_T at the incident angle of 0–80°under (C) Off-state and (D) On-state.

Note S15: To evaluate metasurface performance at varying orientation angles, we conducted oblique-incidence tests. Tests employed the free-space measurement method: the sample was positioned at the center of a copper-foil-covered board, with foil removed directly behind the sample (**Figure S31A**). **Figure S31B** demonstrates that the LIG metasurface maintains functionality as the incident angle varies from 0 ° to 80 °. As exhibited in **Figure S31C** and **S31D**, the average SE_T under Off-state decreases with angle (down to 13.39 dB at 80°), while under On-state it basically increases (up to 1.10 dB at 50 ° and 0.92 dB at 80 °). These results demonstrate that the LIG metasurface enables electromagnetic shielding modulation over a wide range of incident angles.

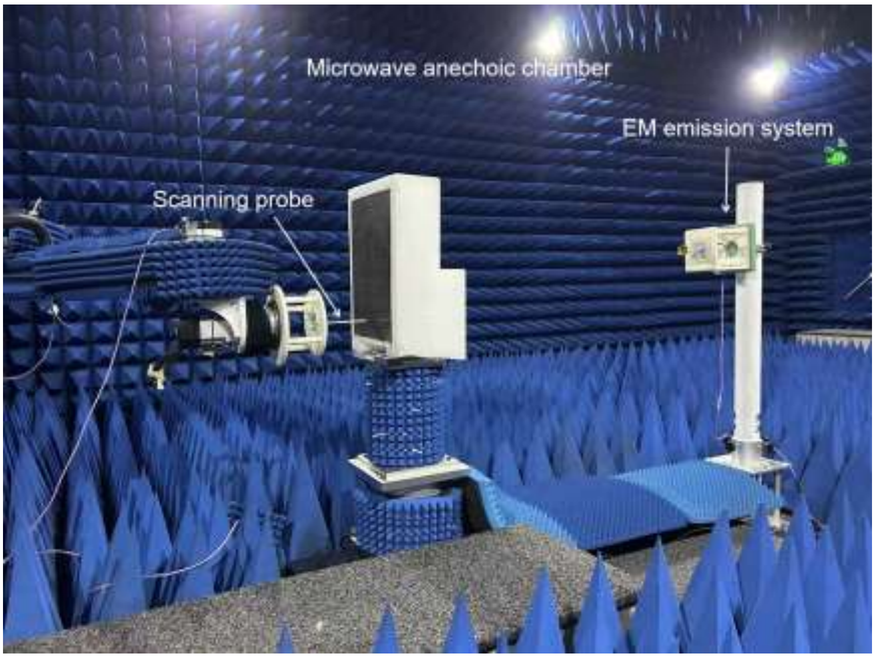

Figure S32. Photograph of near-field scanning test system.

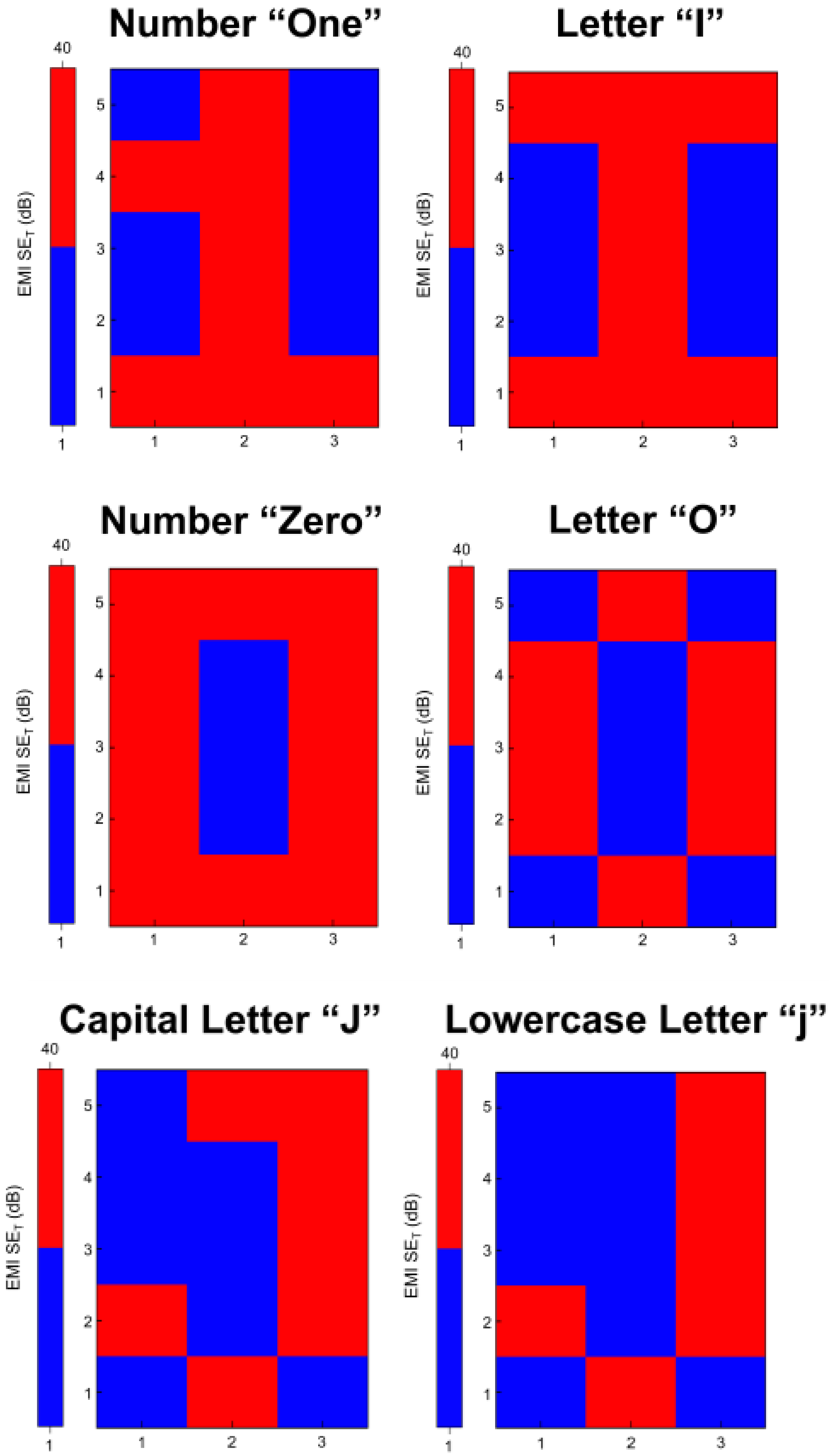

Figure S33. Display of near-field scanning test results of the electromagnetic shielding switch.

Reference:

1. Lee JS, Kim J-W, Lee JH *et al.* Flash-induced high-throughput porous graphene via synergistic photo-effects for electromagnetic interference shielding. *Nano Micro Lett* 2023; **15**: 191.
2. Harito C, Bavykin DV, Yuliarto B *et al.* Inhibition of polyimide photodegradation by incorporation of titanate nanotubes into a composite. *J Polym Environ* 2019; **27**: 1505-15.
3. Srinivasan R. Ablation of polymethyl methacrylate films by pulsed (ns) ultraviolet and infrared (9.17 µm) lasers: A comparative study by ultrafast imaging. *J Appl Phys* 1993; **73**: 2743-50.
4. Musto P, Karasz FE, MacKnight WJ. Fourier transform infra-red spectroscopy on the thermo-oxidative degradation of polybenzimidazole and of a polybenzimidazole/polyetherimide blend. *Polymer* 1993; **34**: 2934-45.
5. DeIasi R, Russell J. Aqueous degradation of polyimides. *J Appl Polym Sci* 1971; **15**: 2965-74.
6. Lin J, Peng Z, Liu Y *et al.* Laser-induced porous graphene films from commercial polymers. *Nat Commun* 2014; **5**: 5714.
7. Inagaki M, Harada S, Sato T *et al.* Carbonization of polyimide film “kapton”. *Carbon* 1989; **27**: 253-7.
8. Schuepfer DB, Badaczewski F, Guerra-Castro JM *et al.* Assessing the structural properties of graphitic and non-graphitic carbons by raman spectroscopy. *Carbon* 2020; **161**: 359-72.
9. Dreyfus RW. Cn temperatures above laser ablated polyimide. *Appl Phys A* 1992; **55**: 335-9.
10. Cheng L, Yeung CS, Huang L *et al.* Flash healing of laser-induced graphene. *Nat Commun* 2024; **15**: 2925.
11. Chen Y, Xie B, Long J *et al.* Interfacial laser-induced graphene enabling high-performance liquid-solid triboelectric nanogenerator. *Adv Mater* 2021; **33**: 2104290.
12. Xu K, Fujita Y, Lu Y *et al.* A wearable body condition sensor system with wireless feedback alarm functions. *Adv Mater* 2021; **33**: 2008701.
13. Xiao H, Li Y, Chen R *et al.* Integrative design of laser-induced graphene array with lithiophilic MnO_x nanoparticles enables superior lithium metal batteries. *eScience* 2023; **3**: 100134.
14. Ling Y, Pang W, Li X *et al.* Laser-induced graphene for electrothermally controlled, mechanically guided, 3D assembly and human-soft actuators interaction. *Adv Mater* 2020; **32**: 1908475.
15. Liu Y, Yuan J, Zhou J *et al.* Laser solid-phase synthesis of graphene shell-encapsulated high-entropy alloy nanoparticles. *Light Sci Appl* 2024; **13**: 270.
16. Sinha K, Meng L, Xu Q *et al.* Laser induction of graphene onto lignin-upgraded flexible polymer matrix. *Mater Lett* 2021; **286**: 129268.
17. Cao L, Zhu S, Pan B *et al.* Stable and durable laser-induced graphene patterns embedded in polymer substrates. *Carbon* 2020; **163**: 85-94.

18. Behrent A, Borggraefe V, Baeumner AJ. Laser-induced graphene trending in biosensors: Understanding electrode shelf-life of this highly porous material. *Analytical and Bioanalytical Chemistry* 2024; **416**: 2097-106.
19. Choi K-H, Park S, Hyeong S-K *et al.* Triboelectric effect of surface morphology controlled laser induced graphene. *J Mater Chem A* 2020; **8**: 19822-32.
20. Ji J, Wang Y, Zhao W *et al.* Laser-induced graphene/ticn on a polyimide/MXene film as interference shielding materials for terahertz electromagnetic waves. *ACS Appl Nano Mater* 2023; **6**: 23401-9.
21. Liu X, Li Y, Sun X *et al.* Off/on switchable smart electromagnetic interference shielding aerogel. *Matter* 2021; **4**: 1735-47.
22. Chen Y, Liu Y, Li Y *et al.* Highly sensitive, flexible, stable, and hydrophobic biofoam based on wheat flour for multifunctional sensor and adjustable EMI shielding applications. *ACS Applied Materials & Interfaces* 2021; **13**: 30020-9.
23. Wang G, Yi D, Jia X *et al.* Structural design of compressible shape-memory foams for smart self-fixable electromagnetic shielding with reduced reflection. *Mater Today Phys* 2022; **22**: 100612.
24. Shen B, Li Y, Zhai W *et al.* Compressible graphene-coated polymer foams with ultralow density for adjustable electromagnetic interference (EMI) shielding. *ACS Appl Mater Interfaces* 2016; **8**: 8050-7.
25. Jia X, Shen B, Zhang L *et al.* Construction of shape-memory carbon foam composites for adjustable emi shielding under self-fixable mechanical deformation. *Chem Eng J* 2021; **405**: 126927.
26. Chen J, Jiang W-j, Zeng Z *et al.* Multifunctional shape memory foam composites integrated with tunable electromagnetic interference shielding and sensing. *Chem Eng J* 2023; **466**: 143373.
27. He YJ, Shao YW, Xiao YY *et al.* Multifunctional phase change composites based on elastic MXene/silver nanowire sponges for excellent thermal/solar/electric energy storage, shape memory, and adjustable electromagnetic interference shielding functions. *ACS Appl Mater Interfaces* 2022; **14**: 6057-70.
28. Wang S, Wang Z, Zheng SY *et al.* Multifunctional heterostructured composite foam with tunable electromagnetic interference shielding. *Compos Sci Technol* 2024; **248**: 110482.
29. Li J, Zhang Y, Li X *et al.* Oriented magnetic liquid metal-filled interlocked bilayer films as multifunctional smart electromagnetic devices. *Nano Res* 2023; **16**: 1764-72.
30. Feng D, Xu D, Wang Q *et al.* Highly stretchable electromagnetic interference (EMI) shielding segregated polyurethane/carbon nanotube composites fabricated by microwave selective sintering. *J Mater Chem C* 2019; **7**: 7938-46.
31. Li P, Du D, Guo L *et al.* Stretchable and conductive polymer films for high-performance electromagnetic interference shielding. *J Mater Chem C* 2016; **4**: 6525-32.
32. Bian J, Zhou X, Zhou X *et al.* High-strain-sensitive dynamically adjustable electromagnetic interference shielding elastomer with pre-linked nickel chains. *Sci China Mater* 2024; **67**: 629-41.

33. Zhu R, Li Z, Deng G *et al.* Anisotropic magnetic liquid metal film for wearable wireless electromagnetic sensing and smart electromagnetic interference shielding. *Nano Energy* 2022; **92**: 106700.
34. Yao B, Hong W, Chen T *et al.* Highly stretchable polymer composite with strain-enhanced electromagnetic interference shielding effectiveness. *Adv Mater* 2020; **32**: 1907499.
35. Yu D, Liao Y, Song Y *et al.* A super-stretchable liquid metal foamed elastomer for tunable control of electromagnetic waves and thermal transport. *Adv Sci* 2020; **7**: 2000177.
36. Liu Z, Wan F, Mou L *et al.* A general approach for buckled bulk composites by combined biaxial stretch and layer-by-layer deposition and their electrical and electromagnetic applications. *Adv Electron Mater* 2019; **5**: 1800817.
37. Zhang M, Zhang P, Wang Q *et al.* Stretchable liquid metal electromagnetic interference shielding coating materials with superior effectiveness. *J Mater Chem C* 2019; **7**: 10331-7.
38. Liao S-Y, Wang X-Y, Huang H-P *et al.* Intelligent shielding material based on VO_2 with tunable near-field and far-field electromagnetic response. *Chem Eng J* 2023; **464**: 142596.
39. Li C, Li D, Zhang M *et al.* Succulent-inspired implicit structural change for smart "on/off" switchable and flexible EMI shielding coating. *ACS Appl Mater Interfaces* 2024; **16**: 12939-50.
40. Liao S-Y, Wang X-Y, Shi Y-Y *et al.* Reversible switching between microwave absorption and emi shielding of VO_2 composite foam. *Small* 2024; **20**: 2402841.
41. Liang S, Guan H, Zhang H *et al.* Tunable high-performance electromagnetic interference shielding of VO_2 nanowires-based composite. *ACS Appl Mater Interfaces* 2024; **16**: 21024-33.
42. Cheng Z, Wang R, Cao Y *et al.* Intelligent off/on switchable microwave absorption performance of reduced graphene oxide/vo_2 composite aerogel. *Adv Funct Mater* 2022; **32**: 2205160.
43. Qian H, Jiang W, shi X *et al.* Pushing electromagnetic interference shielding self-enhanced based on smart $Ti_3C_2T_x$-WVO_2 thermal management composite. *Carbon* 2023; **210**: 118081.
44. Wang Y, Cheng X-D, Song W-L *et al.* Hydro-sensitive sandwich structures for self-tunable smart electromagnetic shielding. *Chem Eng J* 2018; **344**: 342-52.
45. Han M, Zhang D, Shuck CE *et al.* Electrochemically modulated interaction of MXenes with microwaves. *Nature Nanotechnology* 2023; **18**: 373-9.
46. Wen B, Wang X, Zhang Y. Ultrathin and anisotropic polyvinyl butyral/Ni-graphite/short-cut carbon fibre film with high electromagnetic shielding performance. *Compos Sci Technol* 2019; **169**: 127-34.
47. Hong J, Xu P. Electromagnetic interference shielding anisotropy of unidirectional CFRP composites. *Materials* 2021, doi: 10.3390/ma14081907
48. Hong X, Zhong L, Wan J *et al.* Polarization selection characteristics of carbon fiber orientation and interweaving for electromagnetic interference shielding behaviors. *Text Res J* 2021; **92**: 004051752110342.

49. Liu Z, Wang G, Li P *et al.* Gradient in-plane oriented porous carbon inspired by fabrication of toasts for elegant emi shielding performance. *Carbon* 2023; **207**: 136-43.
50. Yoo DK, Woo HC, Jhung SH. Effective removal of particulate matter from air by using zeolite-coated filters. *J Mater Chem A* 2020; **8**: 17960-8.
51. Dai X, Zhou J, Ma Z *et al.* A smart amorphous wire composite with tunable electromagnetic shielding. *Small Struct* 2024; **5**: 2300405.
52. Zeng Z, Wang C, Siqueira G *et al.* Nanocellulose-MXene biomimetic aerogels with orientation-tunable electromagnetic interference shielding performance. *Adv Sci* 2020; **7**: 2000979.
53. Wei Y, Hu C, Dai Z *et al.* Highly anisotropic MXene@wood composites for tunable electromagnetic interference shielding. "*Composites, Part A*" 2023; **168**: 107476.
54. Deng Z, Li L, Tang P *et al.* Controllable surface-grafted MXene inks for electromagnetic wave modulation and infrared anti-counterfeiting applications. *ACS Nano* 2022; **16**: 16976-86.
55. Pinheiro T, Morais M, Silvestre S *et al.* Direct laser writing: From materials synthesis and conversion to electronic device processing. *Adv Mater* 2024; **36**: 2402014.
56. Guo H, Shi Y, Pan F *et al.* Tough, stretchable dual-network liquid metal-based hydrogel toward high-performance intelligent on-off electromagnetic interference shielding, human motion detection and self-powered application. *Nano Energy* 2023; **114**: 108678.
